# Anti-GPIIIa antibody and CD4 count identify an autoimmune-enriched phenotype of HIV-associated thrombocytopenia: development and internal validation of a clinical nomogram

**DOI:** 10.3389/fimmu.2026.1847525

**Published:** 2026-07-07

**Authors:** Xia Liu, Lemin Wen, Zhoulin Zhong, Hangbiao Qiang, Lida Mo, Wenyi Dong, Wen Huang, Shuyu Nong, Zheng Huang, Zhiman Xie, Mei Yu

**Affiliations:** 1 Department of Infection, HIV/AIDS Clinical Treatment Center of Guangxi (Nanning), The Fourth People’ s Hospital of Nanning, Nanning, China; 2 Department of Gastrointestinal Endoscopy, HIV/AIDS Clinical Treatment Center of Guangxi (Nanning), The Fourth People’ s Hospital of Nanning, Nanning, China; 3 Laboratory of Traditional Chinese Medicine Clinical and Translational Medicine, Nanning Hospital of Traditional Chinese Medicine, Nanning, China; 4 Department of Clinical Laboratory, Nanning Blood Center, Nanning, China; 5 Key Laboratory of Infectious Diseases, HIV/AIDS Clinical Treatment Center of Guangxi (Nanning), The Fourth People’ s Hospital of Nanning, Nanning, China; 6 Department of Clinical Laboratory, HIV/AIDS Clinical Treatment Center of Guangxi (Nanning), The Fourth People’ s Hospital of Nanning, Nanning, China

**Keywords:** anti-GPIIIa antibody, CD4 count, clinical prediction model, HIV-associated thrombocytopenia, nomogram, platelet autoimmunity

## Abstract

**Background:**

Thrombocytopenia is a frequent complication of human immunodeficiency virus (HIV) infection, tire arising through diverse mechanisms including autoimmune platelet destruction, advanced immunodeficiency, and systemic comorbidities. Distinguishing thrombocytopenia driven primarily by autoimmune platelet destruction from that related to advanced immunodeficiency remains a clinical challenge, as current standard markers often fail to stratify these distinct pathophysiological phenotypes.

**Methods:**

We conducted a case-control study enrolling 196 HIV-infected individuals (98 thrombocytopenia cases, 98 controls) to develop a clinical prediction model for HIV-associated thrombocytopenia. Candidate predictors were identified through univariable analysis, and AIC-based backward stepwise logistic regression was used to construct a parsimonious multivariable model. A nomogram was generated for bedside risk stratification, and dose-response analysis examined CD4+ T-cell counts and anti-platelet autoimmunity. Model performance was assessed through bootstrap resampling and 10-fold cross-validation, calibration by the Hosmer-Lemeshow test, and clinical utility by Decision Curve Analysis (DCA).

**Results:**

Anti-GPIIIa antibody positivity emerged as the dominant predictor of thrombocytopenia (aOR = 35.3; 95% CI: 9.2–135.6; *P* < 0.001). The final 4-variable model—incorporating anti-GPIIIa antibody, CD4+ count, albumin, and neutrophil count—achieved AUC of 0.862 (95% CI: 0.811–0.913). At the optimal cutoff, the model demonstrated high specificity (96.9%) and positive predictive value (95.6%). Internal validation confirmed robustness, with bootstrap-corrected AUC of 0.857 (optimism = 0.005) and mean cross-validation AUC of 0.867 ± 0.055. Dose-response analysis (CD4 ≤600 cells/μL; n = 167) revealed distinct risk trajectories: anti-GPIIIa-positive patients maintained consistently high thrombocytopenia risk (100% at CD4 <150 cells/μL, declining to approximately 80% at CD4 >300 cells/μL), whereas antibody-negative patients showed progressive risk reduction (from ~58% at CD4 <50 to ~17% at CD4 >380 cells/μL); the CD4 × anti-GPIIIa interaction was not statistically significant (P = 0.427).

**Conclusions:**

This parsimonious model identifies an autoimmune-enriched phenotype of HIV-associated thrombocytopenia, characterized by anti-GPIIIa positivity and reduced immune and hematological reserves. Rather than confirming an immune-mediated etiology, the nomogram supports risk stratification to identify patients warranting further immunological evaluation. As treatment response was not prospectively evaluated and no independent reference standard was applied, external validation and prospective studies are required before clinical implementation.

## 1 Introduction

Thrombocytopenia is one of the most prevalent hematological complications in HIV-infected individuals, historically affecting 30-40% of untreated patients (1, 2). Despite the widespread use of combination antiretroviral therapy (cART), thrombocytopenia persists in 11-16.3% of treated patients, representing a substantial clinical burden given the global HIV prevalence (3, 4).While often asymptomatic, severe thrombocytopenia poses significant risks of hemorrhage, limits the use of necessary medications, and complicates invasive procedures (5–7). The pathogenesis of HIV-associated thrombocytopenia is notably heterogeneous, involving two distinct and often overlapping mechanisms: (1) accelerated peripheral destruction mediated by platelet-specific autoantibodies (a mechanism analogous to primary Immune Thrombocytopenia, ITP) (8–10), and (2) impaired platelet production due to direct viral infection of megakaryocytes (11, 12), drug toxicity (13), or generalized bone marrow suppression (14, 15).

While the detection of platelet-specific autoantibodies strongly suggests an immune etiology, relying solely on antibody status is often insufficient for clinical decision-making (16–18), given the low sensitivity (53%) of current testing methods (16). First, the presence of autoantibodies does not always correlate linearly with the severity of thrombocytopenia (19), as host compensatory mechanisms (e.g., thrombopoiesis) vary with HIV disease stage (14, 15). Second, HIV patients frequently present with “mixed” pathologies where immune destruction coexists with viral suppression or drug toxicity (14, 20), with secondary causes including opportunistic infections and medications (21). Therefore, distinguishing dominant immune-mediated destruction from production failure requires a holistic assessment (22, 23). Currently, clinicians lack an integrated tool that combines immunological markers with hematological indices to accurately stratify the risk of HIV-associated thrombocytopenia in this complex population.

Although the presence of platelet-specific autoantibodies, particularly against glycoproteins (GP) IIb/IIIa or Ib/IX, is a hallmark of the immune-mediated mechanism (17, 24), antibody testing is not universally performed in routine HIV care and is rarely integrated into standardized clinical decision-making algorithms (25, 26). Furthermore, the interaction between host immune status (CD4+ T-cell count) and autoantibody production remains complex (3, 27, 28). While classic HIV progression involves CD4 depletion, the production of autoantibodies often requires a preserved, albeit dysregulated, B-cell response characterized by chronic activation and hypergammaglobulinemia (29, 30), and autoimmune phenomena can paradoxically persist across different CD4 strata (28). Relying solely on retrospective responses to treatment (e.g., defining immune thrombocytopenia by a platelet rise after steroid administration) is impractical for initial diagnostic triage in HIV patients, where multiple overlapping etiologies complicate diagnosis (26). There is an urgent need for a prospective tool that integrates specific immunological markers with routine clinical parameters to stratify thrombocytopenia risk and identify patients who may warrant further immunological evaluation at the time of presentation.

To address this gap, we conducted a case-control study to elucidate the distinct clinical and immunological profiles of patients with HIV-associated thrombocytopenia. Specifically, we investigated the independent predictive value of Anti-GPIIIa antibodies and their interaction with CD4^+^ T-cell counts. Based on these findings, we developed and internally validated a nomogram-based clinical prediction model. This study aims to provide clinicians with a risk-stratification tool to identify patients with an autoimmune-enriched thrombocytopenia phenotype who may warrant further immunological evaluation, with the recognition that external validation and prospective treatment-response studies are required before clinical implementation.

## 2 Materials and methods

### 2.1 Study design and setting

This case-control study was conducted at The Fourth People’s Hospital of Nanning (Guangxi HIV/AIDS Clinical Treatment Center) between November 2024 and June 2025. The study incorporated both prospective and retrospective components: platelet-specific autoantibodies were prospectively measured for all enrolled participants, while other clinical and laboratory data were retrospectively extracted from electronic medical records.

The study protocol was approved by the Ethics Committee of The Fourth People’s Hospital of Nanning (Approval Number: [2025]83) and adhered to the principles of the Declaration of Helsinki. Written informed consent was obtained from all participants prior to antibody testing. Patient confidentiality was maintained through data anonymization throughout the analysis.

### 2.2 Study population and eligibility criteria

The study cohort comprised 196 HIV-infected patients: 98 cases with thrombocytopenia and 98 controls without thrombocytopenia. Controls were randomly selected from HIV-infected patients without thrombocytopenia during the same enrollment period. The two groups were comparable in age and sex distribution (both P > 0.05).

Inclusion criteria were: (1) confirmed HIV-1 infection documented by Western Blot; (2) age ≥18 years; and (3) availability of complete baseline clinical data, including CD4/CD8 T-cell counts and platelet-specific autoantibody profiles (GP Ib, GPIX, GP IIb, GP IIIa, and GMP140).

Case definition: Thrombocytopenia was defined as a platelet count <100×10^9^/L on at least two consecutive measurements within a 4-week interval, in the absence of other identifiable causes.

Control definition: Controls were HIV-infected patients with platelet counts ≥100×10^9^/L, randomly selected from the same clinical service during the enrollment period.

Exclusion criteria were designed to isolate immune-mediated mechanisms: (1) pregnancy; (2) primary hematologic malignancies (e.g., acute leukemia, aplastic anemia, myelodysplastic syndrome); and (3) active opportunistic infections or concurrent malignancies known to cause direct bone marrow suppression (e.g., disseminated mycobacterial infection, lymphoma), unless adequately controlled at the time of enrollment.

A proportion of enrolled patients were newly diagnosed with HIV infection at the time of hospital admission and had not yet initiated antiretroviral therapy (ART) at enrollment, consistent with the inpatient setting of this regional HIV/AIDS treatment center. The differential ART exposure between groups was addressed through pre-specified sensitivity analyses (Section 2.4).

### 2.3 Laboratory assessments and outcome definitions

#### 2.3.1 Primary outcome definition

The primary outcome was HIV-associated thrombocytopenia, defined as a platelet count <100×10^9^/L documented on at least two consecutive measurements within a 4-week period. This diagnosis was established after systematic exclusion of other potential causes of thrombocytopenia, including drug-induced thrombocytopenia, hypersplenism secondary to cirrhosis, primary hematologic disorders, and active opportunistic infections.

#### 2.3.2 Platelet-specific autoantibody quantification (prospective testing)

Platelet-associated autoantibodies were quantified using a multiplex Flow Cytometric Immunobead Assay (Suzhou Yuandeweikang Biomedical Co., Ltd., Jiangsu, China). This assay simultaneously detects IgG antibodies against five distinct platelet surface glycoproteins: GP Ib, GPIX, GP IIb, GP IIIa, and GMP140.

Briefly, patient plasma was incubated with polystyrene beads coated with specific capture antigens. Bound autoantibodies were detected using FITC-conjugated anti-human IgG and analyzed on a flow cytometer (Beckman Coulter, USA). Results were expressed as the Mean Fluorescence Intensity (MFI). Positivity was determined using specific cut-off (C.O.) values derived from the mean fluorescence intensity (MFI) of the negative control group (normal human platelets). In accordance with the manufacturer’s instructions, the cut-off thresholds were calculated as follows: C.O. = 1.9×Mean MFI_negative_ control for Anti-GP Ib, and C.O. = 1.5×Mean MFI_negative_ control for Anti-GP IIIa, Anti-GP IIb, Anti-GP IX, and Anti-GMP 140.

#### 2.3.3 Biomarker selection

All five platelet-specific autoantibodies were assayed. Although both Anti-GP IIIa and Anti-GP IIb were significantly associated with thrombocytopenia, they exhibited high collinearity. Anti-GP IIIa was selected as the representative immunological biomarker for the final multivariate model because it demonstrated the strongest statistical association and ensured model stability.

#### 2.3.4 Routine clinical and laboratory measurements (retrospective data collection)

All routine laboratory tests were performed at the Department of Clinical Laboratory, The Fourth People’s Hospital of Nanning, in accordance with standard operating procedures and quality control protocols. Data were retrospectively extracted from the hospital’s electronic medical record system for tests performed within ±7 days of platelet autoantibody testing.

#### 2.3.5 Hematological parameters

Peripheral venous blood samples were collected in ethylenediaminetetraacetic acid (EDTA) anticoagulant tubes (BD Vacutainer^®^). Complete blood counts, including hemoglobin (HGB), white blood cell count (WBC), platelet count (PLT), absolute neutrophil count, absolute lymphocyte count, and red cell distribution width (RDW), were analyzed using automated hematology analyzers employing impedance and flow cytometry technology (Sysmex XN-9000 Series, Sysmex Corporation, Kobe, Japan; or Mindray BC-6800Plus, Mindray Bio-Medical Electronics Co., Ltd., Shenzhen, China).

#### 2.3.6 Immunological parameters

CD3+, CD4+, CD8+, and CD45+ T-lymphocyte subsets were enumerated using flow cytometry (BD FACSVia™ Flow Cytometer, BD Biosciences, San Jose, CA, USA) with EDTA-anticoagulated whole blood. CD4/CD8 ratios were calculated accordingly.

#### 2.3.7 Biochemical parameters

Serum samples were collected in plain tubes (red-top) or serum separator tubes (yellow-top with gel) and analyzed using Canon TBA-FX series automated biochemical analyzers (Canon Medical Systems Corporation, Tochigi, Japan):

- Albumin: Bromocresol green colorimetric method
- Total bilirubin (TB): Vanadate oxidation method
- Aspartate aminotransferase (AST): Enzymatic rate assay using aspartate substrate
- Ferritin: Immunoturbidimetric assay

#### 2.3.8 Inflammatory markers

C-reactive protein (CRP) was quantified using the Jet-iStar MAX Immunofluorescence Analyzer (Joinstar Biomedical Technology Co., Ltd., Hangzhou, China) via immunofluorescence dry quantitative assay with EDTA-anticoagulated samples.

#### 2.3.9 Coagulation profile

Blood samples for coagulation studies were collected in 0.109 M sodium citrate anticoagulant tubes (citrate-to-blood ratio 1:9). Prothrombin time (PT), activated partial thromboplastin time (APTT), thrombin time (TT), international normalized ratio (INR), and fibrinogen (Fib) were determined using the magnetic bead method on a Stago STA-R Evolution automated coagulation analyzer (Diagnostica Stago, Asnières-sur-Seine, France).

#### 2.3.10 Viral markers

Cytomegalovirus (CMV) DNA was detected in serum samples (collected in yellow-top serum separator tubes) using real-time fluorescence polymerase chain reaction (PCR) on the Amplex Automated Nucleic Acid Purification and PCR Analysis System (Qiagen, Hilden, Germany).

### 2.4 Statistical analysis

Statistical analyses were performed using R software version 4.5.2 (R Foundation for Statistical Computing, Vienna, Austria) and SPSS version 27.0 (IBM Corp, Armonk, NY, USA). Continuous variables were compared using the Student’s t-test or Mann-Whitney U test, and categorical variables using the Chi-square test or Fisher’s exact test. A two-sided P-value < 0.05 was considered statistically significant.

#### 2.4.1 Model development and variable selection

Candidate predictors were identified based on clinical plausibility and univariable analysis (*P* < 0.05). Variables meeting these criteria were entered into a multivariable logistic regression model. To construct a parsimonious and clinically interpretable model, we employed backward stepwise elimination based on the Akaike Information Criterion (AIC) using the MASS R package, which penalizes model complexity more effectively than P-value-based selection and reduces the risk of overfitting.

Following initial backward elimination, systematic model refinement was performed according to predefined criteria: (1) excluding variables with *P* > 0.05 in the multivariable model; (2) when multiple antibodies targeting the same antigen complex were both significant, retaining only the marker with stronger effect size to avoid redundancy; and (3) conducting sensitivity analyses to evaluate whether exclusion of borderline-significant variables substantially impaired model discrimination (assessed by DeLong test for AUC comparison).

The final simplified model’s performance was compared with the initial backward-selected model using the DeLong test to ensure that parsimony did not compromise discriminative ability. The events-per-variable (EPV) ratio was calculated to assess overfitting risk, with EPV >10 considered adequate for model stability.

#### 2.4.2 Model performance and internal validation

Model performance was evaluated according to TRIPOD (Transparent Reporting of a multivariable prediction model for Individual Prognosis Or Diagnosis) guidelines:

1. Discrimination: Assessed by the Area Under the Receiver Operating Characteristic (ROC) Curve (AUC) using the pROC package. An AUC >0.8 was considered excellent. The optimal cutoff value was determined using Youden’s index, prioritizing specificity to minimize false-positive immunosuppressive treatment decisions. Model comparisons were performed using the DeLong test.
2. Internal Validation:
  - Bootstrap resampling (1,000 iterations) was performed to assess optimism and calculate the bootstrap-corrected AUC and 95% confidence intervals.
  - 10-fold cross-validation was conducted to evaluate model stability and generalizability across different data subsets.
  - Bootstrap coefficient stability analysis (1,000 iterations) was performed to assess the robustness of regression coefficients, with relative bias calculated as the percentage of absolute bias to the original coefficient.
  - Validation in this study was restricted to internal validation through the above approaches (bootstrap resampling and 10-fold cross-validation). No independent external validation cohort was available within the present dataset; therefore, external validation in geographically, demographically, and ethnically distinct HIV cohorts represents a critical next step before clinical implementation.
3. Calibration: Evaluated using calibration plots (1,000 bootstrap resamples) to compare predicted vs. observed probabilities, and verified by the Hosmer-Lemeshow goodness-of-fit test (ResourceSelection package) and Brier score.
4. Clinical Utility: Decision Curve Analysis (DCA) was performed using the rmda package to quantify the net benefit of the model across a range of threshold probabilities (5%–95%), compared to “treat all” and “treat none” strategies.
5. Sensitivity Analyses: To evaluate the robustness of the final model against potential confounding, three pre-specified sensitivity analyses were conducted:
  - Age and sex adjustment: Age (continuous, years) and sex (categorical) were added as covariates to the final 4-variable model to assess whether the comparable baseline distributions of age and sex confounded the observed associations.
  - ART status adjustment: ART status (binary, yes/no) was added as a covariate to evaluate potential confounding by antiretroviral therapy exposure.
  - ART duration adjustment: ART duration (continuous, months) was substituted for binary ART status to further assess treatment-related confounding.
  - Stratified analysis: The model was refitted within the ART-treated subgroup (n = 157) to eliminate confounding by differential ART exposure between groups.

Model stability was assessed by comparing odds ratios, confidence intervals, and AUC values between the primary and sensitivity models using the DeLong test.

#### 2.4.3 Dose-response analysis

To elucidate the relationship between immune reconstitution and thrombocytopenia risk, we performed a stratified dose-response analysis using logistic regression. Patients with CD4+ T-cell counts exceeding 600 cells/μL were excluded from this analysis due to sparse data in the upper CD4 range (n = 29 excluded; analytic sample n = 167). First, a logistic regression model incorporating CD4 count, anti-GPIIIa status, and their interaction term (CD4 × anti-GPIIIa) was fitted to formally test whether the effect of CD4 count on thrombocytopenia risk differed by antibody status. Separate logistic regression models were then fitted within each antibody stratum (anti-GPIIIa-positive and anti-GPIIIa-negative) to generate stratum-specific predicted probability curves across the CD4 range (0–600 cells/μL). Ninety-five percent confidence intervals were derived using the Delta method on the logit scale and back-transformed to the probability scale. Observed thrombocytopenia rates were superimposed as empirical data points, calculated by stratifying CD4 count into quantile-based groups within each antibody stratum (up to 5 groups; groups with fewer than 2 observations were excluded), with point size proportional to the number of patients in each group. Figures were generated using the ggplot2 package in R.

### 2.5 Visualization

A static nomogram was generated using the rms package to facilitate individualized risk stratification. The nomogram assigns point scores to each predictor proportional to its regression coefficient, with the strongest predictor assigned the maximum weight of 100 points. Total point scores are converted to predicted thrombocytopenia probabilities.

Additionally, a dose-response curve was created using the ggplot2 package to visualize the relationship between CD4 count and thrombocytopenia probability, stratified by anti-GPIIIa status. The visualization includes 95% confidence bands (shaded regions), observed data points (symbol size proportional to sample size in each CD4 stratum), and a vertical reference line at CD4 = 200 cells/μL marking the WHO AIDS definition threshold.

## 3 Results

### 3.1 Distinct clinical profiles and systemic comorbidities

**Figure 1:**
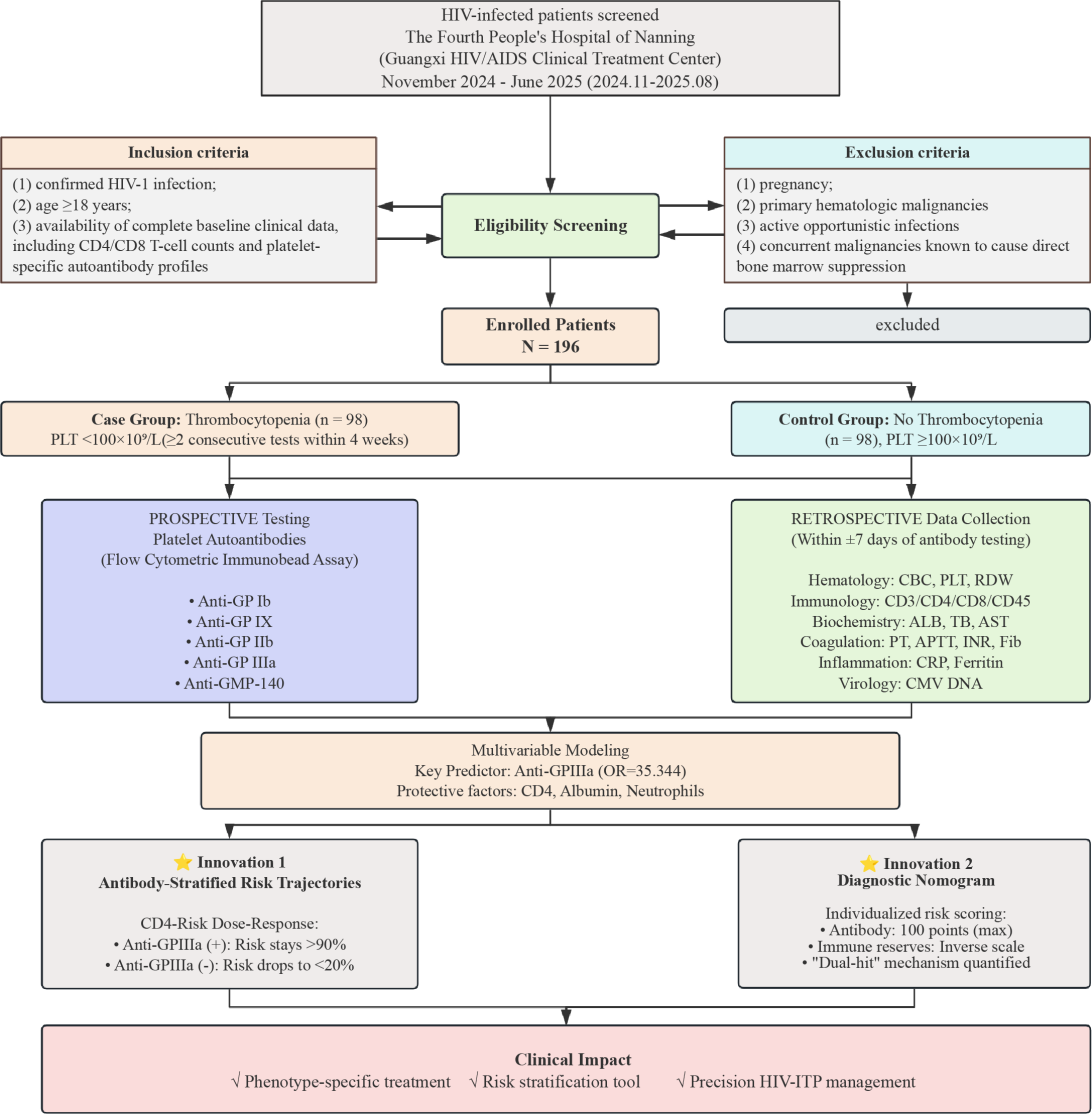
Study profile and analytical framework. The flowchart details the selection and stratification of 196 HIV-infected patients into thrombocytopenia (case) and control cohorts. The workflow integrates platelet autoantibody profiling with clinical parameters to construct a multivariate prediction model and a nomogram for individualized risk stratification. Abbreviations: APTT, activated partial thromboplastin time; CBC, complete blood count; CMV, cytomegalovirus; CRP, C-reactive protein; HIV, human immunodeficiency virus; INR, international normalized ratio; OR, odds ratio; PLT, platelet count; PT, prothrombin time; RDW, red cell distribution width; TB, total bilirubin.

A total of 196 HIV-infected patients were enrolled, comprising 98 cases with thrombocytopenia and 98 controls (**Figure 1**). Baseline characteristics revealed distinct clinical phenotypes (**Table 1**). While age and sex distribution were comparable (*P* > 0.05), patients with thrombocytopenia exhibited significantly compromised nutritional status, evidenced by lower BMI (20.2 vs. 21.8 kg/m², *P* = 0.002). The thrombocytopenia group had a significantly lower proportion of patients receiving ART (70.4% vs. 89.8%, *P* < 0.001) and shorter ART duration among those who had initiated therapy (median 36 months [IQR 0–111] vs. 72 months [IQR 24–120], *P* = 0.016), consistent with the higher proportion of newly diagnosed patients in this group who had not yet initiated treatment at enrollment. In terms of comorbidities, the prevalence of major chronic viral co-infections—specifically Hepatitis B, Hepatitis C, and syphilis—was comparable between the two groups (*P* > 0.05), indicating that these common confounding factors were not the primary drivers of thrombocytopenia in our cohort. However, the thrombocytopenic group presented with significantly higher rates of concurrent hematological abnormalities, including anemia (85.7% *vs.* 64.3%, *P* = 0.001) and leukopenia (29.6% *vs.* 12.2%, *P* = 0.005), as well as active CMV infection (23.5% *vs.* 9.2%, *P* = 0.011), reflecting a more profound state of immune dysregulation and multilineage cytopenia.

**Table 1:** Demographic and clinical characteristics of HIV patients.

Variables	Thrombocytopenia (n = 98)	Non-thrombocytopenia (n = 98)	Test statistic	*P* value
Demographics				
Age, Mean ± SD, years	53.55 ± 12.00	55.76 ± 12.78	t = -0.897	0.371
Female/male sex, n (%)	82 (83.70)/16 (16.30)	74 (75.50)/24 (24.50)	χ²=2.010	0.156
BMI, Mean ± SD, kg/m²	20.16 ± 3.58	21.84 ± 3.81	t = -3.190	0.002
HIV Disease Status				
ART duration, median (IQR), months	36.00 (0,111.00)	72.00 (24.00,120.00)	Z=2.414	0.016
ART, n (%)	69 (70.40)	88 (89.80)	χ²= 11.556	<0.001
Clinical Presentations and Comorbidities				
Anemia	84 (85.70)	63 (64.30)	χ²=12.000	0.001
Leukopenia	29 (29.60)	12 (12.20)	χ²=8.913	0.005
Hematological system neoplasms	2 (2.00)	1 (1.20)	\	1.000
Syphilisa	6 (6.10)	2 (2.00)	\	0.279
Hepatitis B co-infection, n (%)	20 (20.40)	19 (19.40)	χ²=0.032	0.858
Hepatitis C co-infection, n (%)	9 (9.20)	5 (5.10)	χ²=1.231	0.406
CMV infection, n (%)	23 (23.50)	9 (9.20)	χ²=7.320	0.011

BMI, body mass index; HIV, human immunodeficiency virus; ART, antiretroviral therapy; CMV, cytomegalovirus.

### 3.2 Multifaceted hematological and immunological dysregulation

Comparative analysis of laboratory parameters revealed that HIV-associated thrombocytopenia is not an isolated cytopenia but a manifestation of profound systemic dysregulation involving immunological, hematological, and coagulation axes (**Table 2**).

**Table 2:** Univariate analysis of laboratory, immunological, and infection-related variables associated with thrombocytopenia in HIV-infected patients.

Variables	Total (n = 196)	Thrombocytopenia (n = 98)	Non-thrombocytopenia (n = 98)	Test statistic	*P* value
HGB, Mean ± SD	95.22 ± 29.60	85.76 ± 28.76	104.68 ± 27.45	t=-4.71	<.001
Albumin, Mean ± SD	33.86 ± 7.76	30.86 ± 8.19	36.87 ± 5.97	t=-5.87	<.001
WBC, M (Q_1_, Q_3_)	4.90 (3.78, 6.85)	4.40 (2.75, 5.57)	5.70 (4.23, 7.40)	Z=-3.78	<.001
Neutrophils, M (Q_1_, Q_3_)	3.40 (2.20, 4.80)	2.83 (1.70, 4.40)	3.70 (2.50, 4.97)	Z=-3.24	0.001
Lymphocytes, M (Q_1_, Q_3_)	1.00 (0.60, 1.52)	0.80 (0.40, 1.40)	1.20 (0.80, 1.70)	Z=-4.05	<.001
RDW, M (Q_1_, Q_3_)	14.30 (13.00, 17.50)	15.95 (13.53, 18.00)	13.60 (12.60, 14.78)	Z=-4.32	<.001
PT, M (Q_1_, Q_3_)	13.90 (13.00, 15.20)	14.20 (13.50, 16.10)	13.40 (12.80, 14.47)	Z=-4.36	<.001
APTT, M (Q_1_, Q_3_)	38.75 (35.48, 44.20)	40.85 (37.42, 46.43)	36.85 (34.45, 41.45)	Z=-4.68	<.001
INR, M (Q_1_, Q_3_)	1.08 (0.98, 1.20)	1.11 (1.02, 1.30)	1.01 (0.95, 1.13)	Z=-4.37	<.001
TT, M (Q_1_, Q_3_)	18.10 (17.00, 19.40)	18.40 (17.22, 19.88)	17.70 (16.80, 18.50)	Z=-3.82	<.001
Fib, M (Q_1_, Q_3_)	3.49 (2.54, 4.51)	3.37 (2.11, 4.12)	3.65 (2.86, 4.83)	Z=-2.87	0.004
TB, M (Q_1_, Q_3_)	8.05 (5.77, 17.33)	10.05 (6.20, 23.30)	7.25 (5.30, 11.07)	Z=-3.11	0.002
PLT, M (Q_1_, Q_3_)	105.50 (47.00, 216.00)	47.00 (24.00, 74.75)	216.00 (154.75, 259.00)	Z=-12.06	<.001
CD4, M (Q_1_, Q_3_)	209.50 (73.25, 434.25)	120.50 (30.25, 255.25)	344.50 (162.00, 571.75)	Z=-5.37	<.001
CD8, M (Q_1_, Q_3_)	403.00 (214.25, 652.00)	297.00 (174.75, 627.25)	468.50 (281.50, 707.50)	Z=-2.57	0.010
CD4/CD8, M (Q_1_, Q_3_)	0.57 (0.21, 0.94)	0.36 (0.13, 0.83)	0.79 (0.36, 1.12)	Z=-3.90	<.001
CD3, M (Q_1_, Q_3_)	689.50 (372.75, 1219.50)	482.00 (255.50, 870.50)	912.00 (554.75, 1327.00)	Z=-3.98	<.001
CD45, M (Q_1_, Q_3_)	1016.50 (510.50, 1561.00)	780.50 (376.00, 1389.75)	1280.00 (756.75, 1726.75)	Z=-3.53	<.001
AST, M (Q_1_, Q_3_)	29.00 (19.00, 48.50)	36.50 (22.00, 70.00)	24.50 (17.25, 34.75)	Z=-3.58	<.001
CRP, M (Q_1_, Q_3_)	18.35 (5.07, 52.52)	31.30 (7.32, 67.07)	10.10 (5.00, 34.84)	Z=-2.89	0.004
Ferritin, M (Q_1_, Q_3_)	445.90 (202.57, 981.12)	683.10 (286.80, 1109.88)	309.80 (166.40, 642.33)	Z=-3.81	<.001
≥1plateletantibodypositive, n(%)				χ²=51.86	<.001
0	121 (61.73)	36 (36.73)	85 (86.73)		
1	75 (38.27)	62 (63.27)	13 (13.27)		
GPIIIa, n(%)				χ²=55.39	<.001
0	144 (73.47)	49 (50.00)	95 (96.94)		
1	52 (26.53)	49 (50.00)	3 (3.06)		
GPIIb, n(%)				χ²=13.06	<.001
0	158 (80.61)	69 (70.41)	89 (90.82)		
1	38 (19.39)	29 (29.59)	9 (9.18)		
CMV, n(%)				χ²=7.32	0.007
0	164 (83.67)	75 (76.53)	89 (90.82)		
1	32 (16.33)	23 (23.47)	9 (9.18)		

HGB, hemoglobin (g/L); WBC, white blood cell count (×10^9^/L); RDW, red cell distribution width (%); PT, prothrombin time (seconds); APTT, activated partial thromboplastin time (seconds); INR, international normalized ratio (dimensionless); TT, thrombin time (seconds); Fib, fibrinogen (g/L); TB, total bilirubin (μmol/L); PLT, platelet count (×10^9^/L); CD3, CD4, CD8, CD45, T-lymphocyte subsets (cells/μL); CD4/CD8, CD4-to-CD8 ratio (dimensionless); AST, aspartate aminotransferase (U/L); CRP, C-reactive protein (mg/L); Ferritin (μg/L); GPIIIa, glycoprotein IIIa; GPIIb, glycoprotein IIb; CMV, cytomegalovirus. Statistical tests: t, independent samples t-test; Z, Mann-Whitney U test; χ², Chi-square test. Descriptive statistics: SD, standard deviation; M, median; Q_1_, first quartile; Q_3_, third quartile. Antibody coding: 0 = negative (reference); 1 = positive. hemoglobin.

#### 3.2.1 Immunological exhaustion and specific autoimmunity

The thrombocytopenic cohort was characterized by a significantly more severe state of immunodeficiency, evidenced by median CD4^+^ T-cell counts less than half that of controls (120.5 *vs.* 344.5 cells/μL, *P* < 0.001) and a deeply inverted CD4^+^/CD8^+^ ratio (0.36 *vs.* 0.79, *P* < 0.001). Against this background of T-cell depletion, a paradoxically hyperactive B-cell response was observed: platelet-specific autoimmunity emerged as a distinct hallmark. Anti-GPIIIa antibody positivity showed the most dramatic disparity, identified in 50.0% of thrombocytopenic patients compared to only 3.1% of controls (*P* < 0.001). Similarly, the prevalence of anti-GPIIb was significantly elevated (29.6% *vs.* 9.2%, *P* < 0.001), indicating a targeted autoimmune destruction mechanism.

#### 3.2.2 Multilineage cytopenia and hematopoietic stress

Hematological impairment extended beyond the megakaryocytic lineage. Patients with thrombocytopenia exhibited significantly lower hemoglobin levels (85.8 *vs.* 104.7 g/L, *P* < 0.001) and neutrophil counts (2.8 *vs.* 3.7 ×10^9^/L, *P* = 0.001), with a concurrent reduction in total WBC (4.4 *vs.* 5.7 ×10^9^/L, *P* < 0.001) and lymphocytes (0.8 *vs.* 1.2 ×10^9^/L, *P* < 0.001), collectively indicating multilineage hematopoietic suppression. Notably, RDW was significantly elevated in the thrombocytopenia group (16.0% *vs.* 13.6%, *P* < 0.001), reflecting increased anisocytosis and compensatory erythropoietic stress in the setting of systemic inflammation.

#### 3.2.3 Coagulation defects and hepatic dysfunction

A distinct pattern of coagulopathy was evident in the thrombocytopenic group. Global prolongation of coagulation intervals was observed, including PT, APTT, TT, and INR (all *P* < 0.001), accompanied by reduced Fibrinogen levels (3.4 *vs.* 3.7 g/L, *P* = 0.004). This profile, combined with elevated Total Bilirubin (TB, 10.1 *vs.* 7.3 μmol/L, *P* = 0.002) and AST (36.5 *vs.* 24.5 U/L, *P* < 0.001), alongside significantly compromised hepatic synthetic function (albumin 30.9 *vs.* 36.9 g/L, *P* < 0.001), suggests a complex pathophysiology involving hepatic impairment and potential subclinical consumptive coagulopathy.

#### 3.2.4 Systemic inflammation

Finally, these hematological abnormalities occurred within a milieu of heightened systemic inflammation. Inflammatory markers such as CRP (31.3 *vs.* 10.1 mg/L, *P* = 0.004) and, most notably, Ferritin (683.1 *vs.* 309.8 μg/L, *P* < 0.001) were markedly elevated, correlating the severity of thrombocytopenia with the intensity of the systemic inflammatory response.

### 3.3 Identification of independent predictors and construction of the clinical model

Univariable logistic regression analysis (**Supplementary Table 1**) identified 19 variables associated with HIV-associated thrombocytopenia at *P* < 0.10, including platelet-specific autoantibodies (anti-GPIIIa and anti-GPIIb), viral coinfection (CMV), immunological parameters (CD4 count), hematological indices (WBC, hemoglobin, neutrophils, lymphocytes, RDW), coagulation markers (PT, APTT, TT, fibrinogen), liver function tests (TB, AST, albumin), inflammatory markers (CRP), and clinical parameters (BMI, ferritin). Among the platelet-specific autoantibodies, anti-GPIIIa (crude OR 31.67, 95% CI 9.39-106.79, *P* < 0.001) demonstrated a substantially stronger association than anti-GPIIb (crude OR 4.16, 95% CI 1.85-9.35, *P* < 0.001), representing a >7-fold difference in effect size.

All 19 candidate variables, including both anti-GPIIIa and anti-GPIIb, were entered into a multivariable logistic regression model, and backward stepwise elimination based on the Akaike Information Criterion (AIC) was performed (**Supplementary Table 2**). The backward elimination process retained 9 variables— anti-GPIIIa, anti-GPIIb, hemoglobin, neutrophils, lymphocytes, thrombin time, fibrinogen, CD4 count, and albumin — achieving excellent discriminative ability (AUC 0.903, 95% *CI* 0.862-0.945)(**Supplementary Figure 1**). However, this model included three variables with *P* > 0.05 (lymphocytes *P* = 0.054, thrombin time *P* = 0.052, fibrinogen *P* = 0.064) and both platelet-specific autoantibody markers.

To derive a parsimonious final model suitable for clinical implementation, we performed systematic refinement guided by statistical significance, clinical interpretability, and avoidance of redundancy. First, although both anti-GPIIb (adjusted OR [aOR] 3.61, 95% CI 1.12-11.67, *P* = 0.032) and anti-GPIIIa (aOR 39.37, 95% CI 9.17-168.95, *P* < 0.001) were retained by backward elimination, these antibodies target different epitopes of the same platelet integrin αIIbβ3 receptor complex. Given that anti-GPIIIa remained the dominant predictor in multivariable analysis while anti-GPIIb showed markedly attenuated effect compared to its univariable association, we retained only anti-GPIIIa to capture the primary antibody-mediated mechanism while maintaining parsimony and avoiding potential redundancy. Second, variables with *P* > 0.05 in the backward-selected model (lymphocytes, thrombin time, and fibrinogen) were excluded to ensure all retained predictors met conventional significance thresholds. Third, sensitivity analysis comparing models with and without hemoglobin as a potential fifth predictor showed no significant difference in discriminative ability (AUC 0.862 *vs.* 0.864, ΔAUC = 0.002, DeLong *P* = 0.774; **Supplementary Figure 2** and **Supplementary Table 3**). Given hemoglobin’s attenuated significance when evaluated alongside the four core predictors and absence of meaningful contribution to model performance, we selected the more parsimonious 4-variable model for clinical implementation.

The final 4-variable model (**Table 3**) demonstrated good discriminative ability (AUC 0.862, 95% CI 0.811–0.913), representing a modest reduction compared to the 9-variable model (ΔAUC = 0.041, DeLong *P* = 0.012; **Supplementary Figure 3**). However, the simplified model offers superior clinical usability and improved events-per-variable ratio (24.5 *vs.* 10.9), reducing the risk of model overfitting while maintaining all statistically significant predictors. Anti-GPIIIa antibody positivity emerged as the dominant predictor (aOR 35.344, 95% CI 9.208-135.665, *P* < 0.001), exhibiting substantially greater magnitude than the three physiological protective factors: CD4 count (aOR 0.821, 95% CI 0.685-0.984, *P* = 0.033), neutrophils (aOR 0.798, 95% CI 0.683-0.932, *P* = 0.004), and albumin (aOR 0.909, 95% CI 0.854-0.968, *P* = 0.003).

**Table 3:** Final multivariable logistic regression model for predicting HIV-associated thrombocytopenia.

Variable	Beta	OR	CI_Lower	CI_Upper	*P*_Value
(Intercept)	4.045	57.095	6.443	505.963	0.0003
GPIIIa^†^	3.565	35.344	9.208	135.665	<0.001
CD4_scaled^‡^	-0.197	0.821	0.685	0.984	0.033
Albumin (g/L)	-0.095	0.909	0.854	0.968	0.0029
Neutrophils (10^9^/L)	-0.225	0.798	0.683	0.932	0.0044

†Anti-GPIIIa: 0=negative (reference), 1= positive; ‡CD4 count per 100 cells/μL increase. Model performance: AUC = 0.862 (95% CI 0.811–0.913). Abbreviations: CD4_scaled, CD4+ T-cell count (per 100 cells/μL); GPIIIa, glycoprotein IIIa (anti-platelet antibody); AUC, area under the curve; CI, confidence interval; OR, odds ratio; β, regression coefficient. Note: This model was derived from 19 candidate variables through backward stepwise elimination (AIC) followed by systematic refinement. See **Supplementary Tables 1-3** and **Supplementary Figure 1** for detailed model development process.

### 3.4 Model Performance, calibration, and clinical utility

#### 3.4.1 Discriminative performance

**Figure 2:**
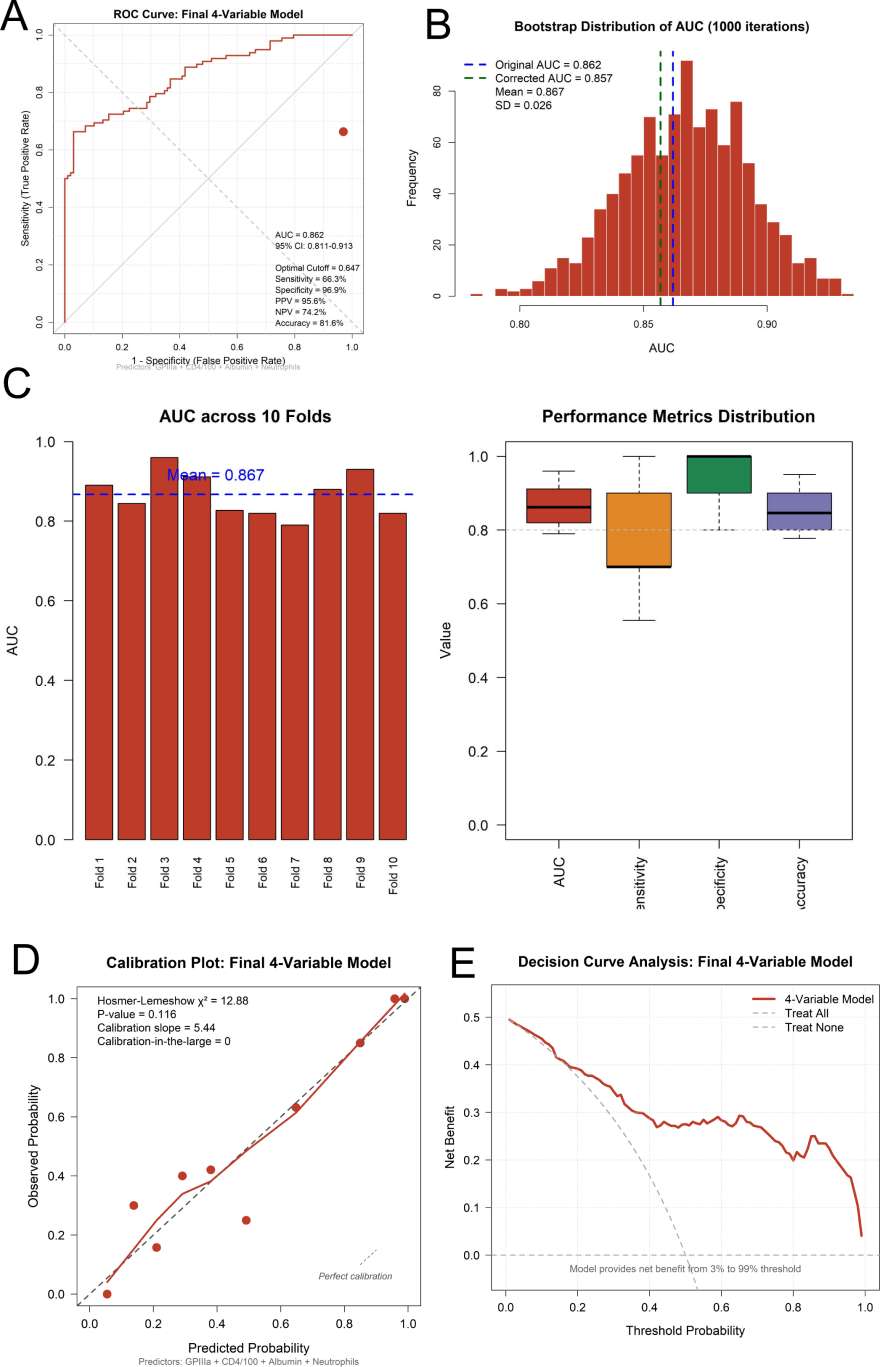
Model performance, validation, and clinical utility. **(A)** ROC Curve. Discriminative performance of the 4-variable prediction model. **(B)** Bootstrap Validation. Distribution of AUC values from 1,000 bootstrap iterations. **(C)** Ten-Fold Cross-Validation. Left panel shows AUC across 10 folds; right panel shows distribution of performance metrics across folds. **(D)** Calibration Plot. Agreement between predicted probabilities and observed frequencies. **(E)** Decision Curve Analysis. Net benefit of using the prediction model compared to treating all or treating none strategies. Abbreviations: AUC, area under the curve; ROC, receiver operating characteristic.

The final simplified 4-variable clinical model (GPIIIa, CD4 count, albumin, and neutrophil count) maintained robust discriminative power despite the substantial reduction in predictors from the backward stepwise model. The Area Under the ROC Curve (AUC) was 0.862 (95% CI 0.811–0.913) (**Figure 2A**), demonstrating excellent diagnostic accuracy. While statistically lower than the backward stepwise model with 9 variables (AUC 0.903, DeLong test *P* = 0.012) (**Supplementary Figure 3**), the simplified model offered superior parsimony and clinical applicability without compromising essential predictive capacity.

At the optimal cutoff value of 0.647 derived from Youden’s index on the ROC curve, the model was calibrated to prioritize specificity, achieving a specificity of 96.9% and a sensitivity of 66.3%. This high specificity indicates that the model is particularly effective at identifying an autoimmune-enriched thrombocytopenia phenotype when positive, with a positive predictive value (PPV) of 95.6%, thereby reducing the likelihood of misclassification in patients with other causes of thrombocytopenia. The negative predictive value (NPV) was 74.2%, and the overall accuracy was 81.6% (**Figure 2A**).

#### 3.4.2 Internal validation

Internal validation through bootstrap resampling (1,000 iterations) confirmed minimal optimism (0.005), with a bootstrap-corrected AUC of 0.857 (95% bootstrap CI 0.815–0.916, SD = 0.026) (**Figure 2B**). The bootstrap distribution of AUC values demonstrated excellent stability, with the bootstrap mean AUC (0.867) closely aligned with the original AUC (0.862), indicating robust generalizability. Notably, all bootstrap iterations yielded AUC values above 0.78, with 95% of values concentrated between 0.82 and 0.92 (**Supplementary Table 4**), confirming consistent discriminative performance across diverse resampling scenarios.

Ten-fold cross-validation further demonstrated stable performance across all folds (**Figure 2C**), with mean AUC of 0.867 ± 0.055 (range 0.79–0.96), mean sensitivity of 75.6% ± 9.5% (range 55.6–100%), mean specificity of 95.0% ± 4.1% (range 80.0–100%), and mean accuracy of 85.2% ± 3.6% (range 77.8–95.0%) (**Supplementary Table 5**). The low variability across folds confirms the model’s stability and lack of overfitting.

Bootstrap analysis of coefficient stability (R = 1,000) revealed that all four predictors maintained stable coefficients with 95% confidence intervals not crossing zero (**Supplementary Table 4**). In terms of relative bias (absolute bias as a percentage of the original coefficient), albumin demonstrated the highest stability (absolute bias = −0.003, relative bias = 3.0%), followed by neutrophil count (absolute bias = −0.012, relative bias = 5.5%) and CD4 count (absolute bias = −0.011, relative bias = 5.6%). GPIIIa showed greater variability (absolute bias = 1.056, relative bias = 29.6%, SD = 3.606, skewness = 3.87) (**Supplementary Table 4**) due to quasi-complete separation in approximately 5% of bootstrap samples, reflecting the exceptionally high prevalence of thrombocytopenia among GPIIIa-positive patients (94.2%, 49/52) compared to GPIIIa-negative patients (34.0%, 49/144). Despite this increased variability, GPIIIa maintained a consistently positive effect across all bootstrap resamples, with 95% confidence intervals (2.71–19.92) entirely above zero, confirming its robust predictive value.

#### 3.4.3 Calibration

The simplified 4-variable model demonstrated acceptable calibration across most probability ranges (**Figure 2D**). The Hosmer-Lemeshow goodness-of-fit test yielded a non-significant result (χ²=12.88, df=8, *P* = 0.116), indicating no statistically significant deviation from perfect calibration, and the Brier score of 0.146 confirmed good overall predictive accuracy. The calibration curve showed reasonable agreement with the ideal 45° reference line, though with some deviation in the mid-probability range (0.3–0.6) where the model slightly overestimated risk. Importantly, the 4-variable model achieved calibration performance comparable to the more complex 9-variable backward model (Hosmer-Lemeshow χ²=6.75, *P* = 0.563), with both models showing similar calibration characteristics (detailed comparison in **Supplementary Figure 4** and **Supplementary Table 6**). This comparability supports the selection of the parsimonious model for clinical implementation without compromising prediction accuracy.

#### 3.4.4 Clinical utility

Decision Curve Analysis (DCA) (**Figure 2E**) demonstrated that using the prediction model provides superior net benefit compared to treating all patients or treating no patients across a wide range of threshold probabilities (approximately 5%–95%). The model showed particularly strong net benefit in the clinically relevant threshold probability range of 20%–60%, where treatment decisions are most uncertain. At a threshold probability of 30%, corresponding to moderate clinical suspicion, the model provided a net benefit of 0.344, translating to approximately 34 net true positives per 100 patients at this threshold (**Supplementary Table 7**). The net benefit remained positive across nearly the entire probability spectrum, confirming the model’s clinical utility across a wide range of decision thresholds.

#### 3.4.5 Sensitivity analyses for potential confounding

Given that ART exposure differed significantly between groups (Section 3.1) and may affect immune reconstitution, marrow function, and platelet recovery, we performed three pre-specified sensitivity analyses to evaluate whether ART status or duration confounded the associations between the four core predictors and thrombocytopenia (**Supplementary Table 8**).

*Covariate-adjusted analyses* Sequential addition of ART status (binary, yes/no) and ART duration (continuous, months) to the final 4-variable model yielded virtually identical estimates for all core predictors. With ART status added, the odds ratios for anti-GPIIIa, CD4 count (per 100 cells/μL), albumin, and neutrophil count were 35.48, 0.83, 0.91, and 0.80, respectively — values that differed from the primary model by less than 2% on the OR scale and that retained statistical significance (all *P* < 0.05). Substituting ART duration for ART status produced equivalent estimates (anti-GPIIIa OR 35.87; CD4 OR 0.83; albumin OR 0.91; neutrophils OR 0.80). Critically, neither ART status (OR 0.66, 95% CI 0.24–1.86, *P* = 0.435) nor ART duration (OR 1.00 per month, 95% CI 0.99–1.00, *P* = 0.617) was independently associated with thrombocytopenia after adjustment, indicating that the prognostic information conveyed by ART exposure is largely captured by its downstream biological consequences—namely, CD4 reconstitution, hepatic synthetic function, and myeloid output(**Supplementary Table 8****).**

*Stratified analysis in ART-treated patients.* To further eliminate confounding by ART exposure, the model was refitted within the ART-treated subgroup (n = 157), a clinically homogeneous population that comprises the majority of patients managed under contemporary “test-and-treat” policies. All four core predictors remained statistically significant with effect sizes closely paralleling the primary model: anti-GPIIIa (OR 32.55, 95% CI 9.44–162.44, *P* < 0.001), CD4 count (per 100 cells/μL, OR 0.81, 95% CI 0.66–0.98, *P* = 0.045), albumin (OR 0.90, 95% CI 0.83–0.97, *P* = 0.006), and neutrophil count (OR 0.81, 95% CI 0.67–0.95, P = 0.014). Model discrimination remained excellent, with an AUC of 0.850 (95% CI 0.790–0.911) representing only a 0.012 decrement compared with the full cohort(**Supplementary Table 9****).**

*Exploratory analysis in ART-naïve patients* In the ART-naïve subgroup (n = 39), the multivariable model could not be reliably fitted due to quasi-complete separation, which arises when one predictor near-perfectly discriminates outcomes within a small sample. This statistical phenomenon precluded formal multivariable inference within this subgroup(**Supplementary Table 9**, Part D), and the limited sample size further limits any interpretation. Findings from this subgroup should therefore be regarded as exploratory and warrant confirmation in larger ART-naïve cohorts.

Adjustment for age and sex. To confirm that the comparable baseline distributions of age and sex did not confound the observed associations, we added age (continuous, years) and sex (categorical) as covariates to the final 4-variable model. All four core predictors retained statistical significance with virtually unchanged effect sizes: anti-GPIIIa (OR 35.19, 95% CI 9.05–136.90, *P* < 0.001), CD4 count (OR 0.824, 95% CI 0.688–0.987, *P* = 0.036), albumin (OR 0.909, 95% CI 0.853–0.968, *P* = 0.003), and neutrophil count (OR 0.800, 95% CI 0.684–0.936, *P* = 0.005). Neither age (OR 0.989, 95% CI 0.961–1.018, *P* = 0.452) nor sex (OR 1.101, 95% CI 0.461–2.626, *P* = 0.829) was independently associated with thrombocytopenia after adjustment. Model discrimination was virtually unchanged (AUC 0.864 vs. 0.862; ΔAUC = 0.002, DeLong *P* = 0.529), confirming that these demographic variables do not confound the primary findings (**Supplementary Figure 5**; **Supplementary Table 10****).**

Collectively, these convergent analyses — encompassing adjustment for age, sex, ART status, ART duration, and stratification by ART treatment — provide robust evidence that the four-variable model is not materially confounded by demographic or treatment-related factors, supporting its stability and applicability across the spectrum of HIV-associated thrombocytopenia.

### 3.5 Distinct risk profiles by antibody status

**Figure 3:**
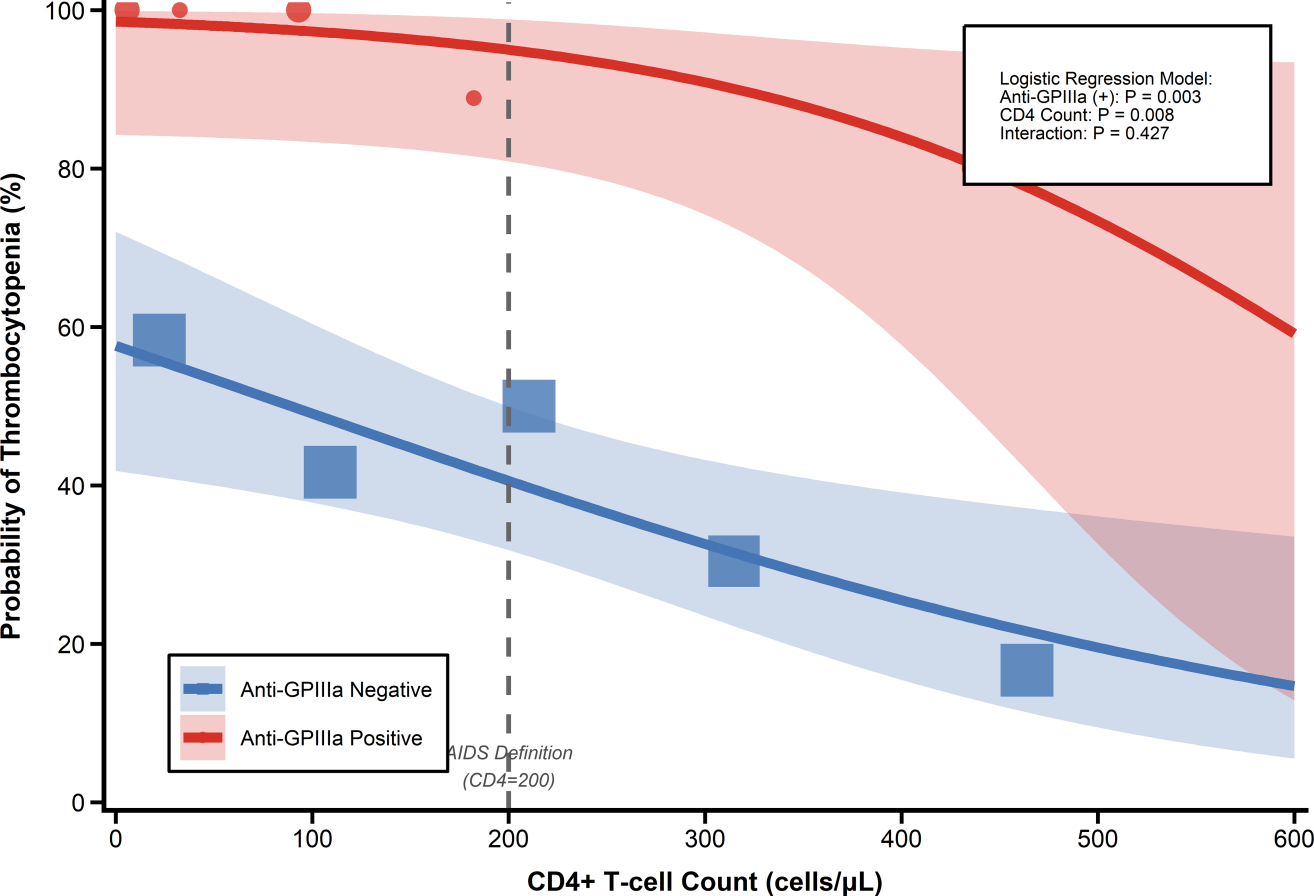
Dose-response relationship between CD4+ T-cell count and thrombocytopenia risk stratified by anti-GPIIIa antibody status. Predicted probability of thrombocytopenia across the CD4+ T-cell count spectrum, stratified by anti-GPIIIa antibody status. The red line represents antibody-positive patients, showing persistently high risk with an attenuated decline across the observed CD4 range. The blue line represents antibody-negative patients, demonstrating progressive risk reduction with immune reconstitution. Shaded areas indicate 95% confidence intervals. The vertical dashed line marks the AIDS-defining threshold (CD4 = 200 cells/μL). Box sizes reflect sample distribution across CD4 strata. Abbreviation: Anti-GPIIIa, anti-glycoprotein IIIa antibody.

To elucidate the relationship between immune reconstitution and thrombocytopenia risk, we modeled the dose–response relationship between CD4+ T-cell count and thrombocytopenia probability, stratified by anti-GPIIIa antibody status (**Figure 3**). In the model including CD4 count, anti-GPIIIa status, and their interaction term, both CD4 count (β = −0.0034 per cell/μL, corresponding to OR = 0.71 per 100 cells/μL increase; P = 0.008) and anti-GPIIIa positivity (β = 3.92, OR = 50.5; *P* = 0.003) were independently associated with thrombocytopenia risk (**Supplementary Table 11**).

Patients with anti-GPIIIa antibodies (red trajectory) demonstrated consistently high predicted risk across the observed CD4 range, with an attenuated decline as CD4 increased and widening uncertainty at higher CD4 counts. This pattern suggests that antibody-mediated platelet destruction may remain a dominant mechanism even with partial immune reconstitution.

In contrast, antibody-negative patients (blue trajectory) exhibited a clear dose-dependent reduction in predicted risk, decreasing from approximately 55% at CD4 <50 cells/μL to approximately 20% at CD4 ≈500 cells/μL. This finding is consistent with thrombocytopenia driven, at least in part, by immunodeficiency-related mechanisms (e.g., impaired megakaryopoiesis and dysregulated cytokine milieu) that may improve with ART-mediated immune restoration.

Although the CD4 × anti-GPIIIa interaction was not statistically significant (*P* = 0.427), the two predicted risk curves were substantially separated across the observed CD4 range (**Figure 3**). Consistently, in the analytic sample restricted to CD4 ≤600 cells/μL (n = 167), thrombocytopenia prevalence was 93.8% in anti-GPIIIa–positive patients versus 39.5% in anti-GPIIIa–negative patients (**Supplementary Table 11**). Collectively, these findings support the clinical utility of anti-GPIIIa testing for identifying an autoimmune-enriched thrombocytopenia phenotype, which may warrant different clinical evaluation from immunodeficiency-associated thrombocytopenia. As treatment response was not assessed in the present study, the therapeutic implications of this distinction require prospective evaluation.

### 3.6 Hierarchical risk contribution and development of a diagnostic nomogram

**Figure 4:**
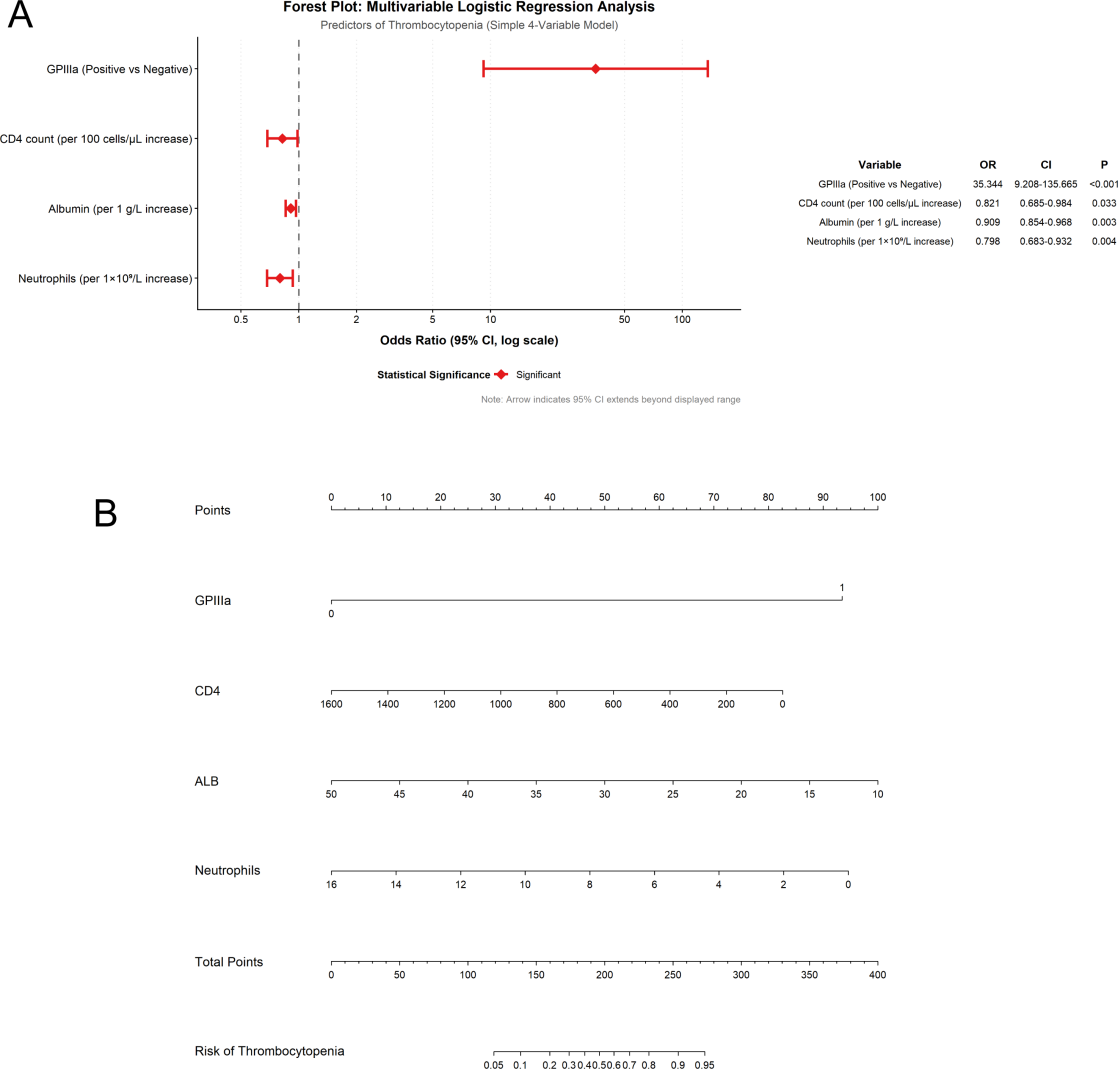
Nomogram and forest plot for the 4-variable prediction model. **(A)** Forest Plot. Odds ratios and 95% confidence intervals from multivariable logistic regression analysis. Anti-GPIIIa antibody positivity showed the strongest association with thrombocytopenia (OR 35.344). **(B)** Nomogram. A point-based scoring system for predicting individual patient risk. Each predictor is assigned points based on its value, and the sum of points corresponds to the predicted probability of thrombocytopenia on the bottom scale. Abbreviations: Anti-GPIIIa, anti-glycoprotein IIIa antibody; CI, confidence interval; OR, odds ratio.

To bridge the gap between statistical significance and clinical utility, we visualized the predictor hierarchy and translated the multivariable model into a pragmatic diagnostic tool (**Figure 4**). The forest plot (**Figure 4A**) visually quantifies the striking disparity in effect sizes among predictors. While physiological reserves—specifically CD4 count, albumin, and neutrophils—functioned as protective factors (OR < 1.0), anti-GPIIIa positivity emerged as the dominant risk factor with an adjusted OR of 35.344 (95% CI 9.208–135.665) in the primary 4-variable model. This necessitated a logarithmic scale to accommodate the disproportionate magnitude of the autoimmune driver compared to physiological markers.

This statistical hierarchy was operationalized into a nomogram (**Figure 4B**) for individualized risk assessment. Consistent with the high odds ratio, the presence of anti-GPIIIa antibody was assigned the maximum weight (100 points) on the scoring scale. The continuous variables (CD4 count, albumin, and neutrophils) were scaled inversely, with lower levels of these physiological parameters corresponding to higher risk points. This inverse scaling visually reinforces the biological premise that HIV-associated thrombocytopenia results from a “dual-hit” mechanism: (1) platelet-destructive autoantibodies establish a high baseline risk that appeared relatively less attenuated by immune reconstitution, and (2) depletion of immune and hematological reserves further amplifies this risk in susceptible individuals. Clinically, this tool enables rapid risk stratification to identify patients who may warrant further immunological evaluation. Notably, as illustrated in **Figure 3**, anti-GPIIIa-positive patients maintained consistently high thrombocytopenia risk across most of the observed CD4 range, suggesting that immune reconstitution alone may be insufficient to resolve thrombocytopenia risk in this subgroup, though prospective studies evaluating treatment response are needed to confirm this observation tire.

## 4 Discussion

Thrombocytopenia in HIV infection represents a diagnostic conundrum, often presenting as a complex interplay of immune-mediated destruction, viral-induced myelosuppression, and diminished physiological reserves (14, 31, 32). While the identification of platelet-specific autoantibodies suggests an ITP-like mechanism (33–35), the clinical translation of this finding has been hindered by the lack of integrated predictive tools that simultaneously account for immunological status and hematological reserves. In this study, we developed and internally validated a nomogram to identify an autoimmune-enriched thrombocytopenia phenotype in HIV patients by integrating serological markers (anti-GPIIIa antibodies) with immunological and hematological parameters. Our 4-variable clinical model, anchored by anti-GPIIIa status and CD4+ count, demonstrated robust discriminative power (AUC 0.862; 95% CI: 0.811–0.913) with a sensitivity of 66.3% and specificity of 96.9% at the optimal cutoff, demonstrating high specificity for identifying an autoimmune-enriched phenotype when positive. We observed a pattern consistent with a “dual-hit” mechanism, where anti-GPIIIa positivity was associated with markedly elevated thrombocytopenia risk, and depletion of physiological reserves (CD4, albumin, and neutrophils) further amplified this risk. while This model transcends conventional binary antibody testing (8), offering a quantifiable, risk-stratified probabilistic tool that integrates both the immunopathological driver and host compensatory capacity, thereby supporting risk stratification to identify patients who may warrant further immunological evaluation. As treatment response was not prospectively evaluated, therapeutic implications require confirmation in prospective studies.

### 4.1 Innovation 1: distinct risk profiles by antibody status—relatively attenuated CD4 dose-response in anti-GPIIIa-positive patients

A pivotal finding of this study is the distinct risk trajectory in anti-GPIIIa-positive patients. Our dose-response analysis demonstrated two strikingly divergent risk trajectories stratified by antibody status (**Figure 3**).

In patients lacking anti-platelet autoantibodies (antibody-negative group), the risk of thrombocytopenia exhibited a strong dose-dependent relationship with CD4+ T-cell counts (β = -0.0034 per cell/μL, OR = 0.71 per 100 cells/μL increase; *P* = 0.008). Thrombocytopenia probability declined progressively from approximately 55% at CD4 <50 cells/μL to below 20% at CD4 >400 cells/μL, reflecting mechanisms driven by advanced HIV-associated marrow exhaustion (31, 36, 37) or viral cytopathic effects on megakaryocytes (38, 39). This CD4-dependent pattern is consistent with previous epidemiological findings demonstrating that thrombocytopenia prevalence increases from 4.8% at CD4 >500 cells/μL to 21.7% at CD4 <200 cells/μL (40), and suggests that immunodeficiency-related thrombocytopenia may be reversible with ART-mediated immune restoration. In striking contrast, patients with anti-GPIIIa antibodies exhibited consistently high predicted risk across the observed CD4 range, with observed rates declining modestly from 100% at CD4 <150 cells/μL to 80% at CD4 >300 cells/μL, displaying a characteristic plateau pattern (**Figure 3**). This pattern suggests that thrombocytopenia risk in anti-GPIIIa-positive patients may be relatively less attenuated by CD4 reconstitution compared with antibody-negative patients. Although the non-significant interaction term (*P* = 0.427) precludes definitive conclusions about true statistical independence, the strong main effect of anti-GPIIIa antibody positivity (*P* = 0.003) and the clinically substantial separation between risk curves across all CD4 strata—with an absolute risk difference exceeding 50 percentage points (93.8% *vs.* 39.5% prevalence in antibody-positive vs. antibody-negative groups) together indicate that anti-GPIIIa-positive patients maintained markedly elevated thrombocytopenia risk across the full observed CD4 range, supporting the clinical utility of anti-GPIIIa testing for risk stratification and suggesting that the relationship between immune reconstitution and platelet survival may differ by antibody status—a hypothesis warranting evaluation in larger prospective studies. This observation is consistent with emerging evidence that immune reconstitution via ART alone may not be sufficient to resolve HIV-associated thrombocytopenia in all patients (41). This finding raises the possibility that anti-GPIIIa-mediated platelet destruction may be relatively less responsive to CD4 recovery, though formal effect modification was not demonstrated and longitudinal studies are needed to confirm this hypothesis.

This observation is supported by the “molecular mimicry” hypothesis, wherein HIV-GPIIIa cross-reactivity may establish autoimmune responses that persist independently of CD4+ reconstitution (9, 42). Li et al. (9) demonstrated through phage display technology that HIV-1 proteins (nef, gag) share structural similarity with platelet GPIIIa49–66 epitopes, and antibodies generated against these HIV mimotopes can induce platelet lysis and thrombocytopenia *in vivo*. Importantly, this autoantibody-mediated platelet destruction mechanism is complement-independent (14), suggesting that once initiated, it may form an autonomous pathological pathway. Our data corroborate this hypothesis: even at CD4 counts >200 cells/μL, anti-GPIIIa-positive patients maintained high thrombocytopenia rates (80–89%), while antibody-negative patients showed significant risk reduction with CD4 recovery (approximately 55% to below 20%).This aligns with observations by Marks et al. (7) that thrombocytopenic patients in the cART era have significantly higher CD4 counts than in earlier studies. Furthermore, Zetterberg et al. (43) demonstrated in the SMART study that ART discontinuation can induce platelet decline even during viral suppression, supporting the concept that autoimmune mechanisms can persist independently of viral load and CD4 levels.

Clinically, these findings suggest that anti-GPIIIa status may provide useful information for risk stratification in the evaluation of HIV-associated thrombocytopenia. Anti-GPIIIa-positive patients maintained markedly elevated thrombocytopenia risk across the observed CD4 range, suggesting that immune reconstitution alone may be insufficient in this subgroup and that further immunological evaluation may be warranted. Whether targeted immunomodulatory interventions are beneficial in this population requires prospective evaluation, as treatment response was not assessed in the present study; existing data suggest that HIV-associated thrombocytopenia can be refractory and prone to relapse, underscoring the need for prospective studies to define optimal management strategies (44, 45).

### 4.2 Innovation 2: hierarchical risk contribution and development of a diagnostic nomogram

The second major contribution of this study is the quantification of hierarchical risk contributions and translation of these findings into a clinically actionable diagnostic tool.

#### 4.2.1 Dominance of the autoimmune mechanism

Our multivariable analysis revealed a striking asymmetry in effect sizes among predictors (**Figure 4A**). Anti-GPIIIa antibody positivity emerged as the overwhelmingly dominant predictor with an adjusted OR of 35.344 (95% CI 9.208–135.665, *P* < 0.001)—a magnitude so disproportionate that logarithmic scaling was required for visualization. In contrast, the physiological reserves functioned as protective factors with comparatively modest effect sizes: CD4 count (aOR 0.821 per 100 cells/μL, *P* = 0.033), albumin (aOR 0.909 per g/L, *P* = 0.003), and neutrophil count (aOR 0.798 per 10^9^/L, *P* = 0.004).

This hierarchy has important mechanistic implications. While immune reconstitution (CD4), nutritional status (albumin), and hematopoietic reserve (neutrophils) all modulate thrombocytopenia risk, they operate as secondary modulators in the presence of the primary autoimmune driver. The significance of albumin, in particular, may reflect the intersection of nutritional status and hepatic thrombopoietin synthesis (46, 47), suggesting that systemic factors contribute to the clinical manifestation of autoimmune thrombocytopenia. Interestingly, while anti-GPIIb was also elevated in univariable analysis (crude OR 4.16), our multivariate modeling prioritized anti-GPIIIa as the more robust predictor. This distinction is clinically relevant, as GPIIIa (CD61) is a major target of pathogenic antibodies affecting both platelet clearance and megakaryocyte maturation (48), and the >7-fold difference in effect size (crude OR 31.67 *vs.* 4.16) supports GPIIIa as the dominant pathogenic epitope in HIV-associated autoimmune thrombocytopenia.

#### 4.2.2 The “dual-hit” model and nomogram implementation

This statistical hierarchy was operationalized into a nomogram (**Figure 4B**) for individualized risk assessment at the bedside. Consistent with its dominant odds ratio, anti-GPIIIa antibody positivity was assigned the maximum weight of 100 points on the scoring scale. The continuous variables (CD4 count, albumin, and neutrophils) were scaled inversely, with lower levels corresponding to higher risk points. This inverse scaling visually reinforces the biological premise of a “dual-hit” mechanism: (1) platelet-destructive autoantibodies establish a high baseline risk that appeared relatively less attenuated by immune reconstitution, and (2) depletion of immune and hematological reserves further amplifies this risk in susceptible individuals.

At the optimal cutoff value of 0.647 derived from Youden’s index, the model was calibrated to prioritize specificity (96.9%) while maintaining acceptable sensitivity (66.3%). This design choice reflects the clinical imperative to minimize false-positive identification of an autoimmune-enriched phenotype, thereby reducing unnecessary escalation to further immunological workup or empirical immunosuppressive treatment in patients with alternative etiologies (e.g., drug toxicity, myelodysplasia, thrombotic microangiopathy). The high positive predictive value (95.6%) indicates that the model demonstrates high specificity for identifying an autoimmune-enriched phenotype when positive, which is critical in resource-limited settings where distinguishing autoimmune destruction from opportunistic infections invading the marrow is essential to support appropriate further evaluation and avoid empirical treatment errors in this complex population (4, 49).

### 4.3 Translational perspective: clinical utility of the nomogram for HIV-associated thrombocytopenia risk stratification in HIV patients

From a translational perspective, our model addresses a critical unmet need in HIV care: integrating immunological biomarkers and routine clinical parameters into a nomogram-based risk-stratification tool to identify patients with an autoimmune-enriched thrombocytopenia phenotype who may warrant further immunological evaluation tire.

The Decision Curve Analysis (DCA) demonstrated a clear net benefit over “treat-all” or “treat-none” strategies across a wide range of threshold probabilities (5%–95%). In the clinically relevant threshold range of 20%–60%, where treatment decisions are most uncertain, the model showed particularly strong performance. At a threshold probability of 30%, the model provided a net benefit of 0.344 compared to 0.286 for the treat-all strategy, representing a net gain equivalent to identifying approximately 5.8 additional true positives per 100 patients after accounting for the harms of unnecessary treatment—reflecting the model’s ability to maintain superior diagnostic yield while substantially reducing overtreatment in antibody-negative patients. Unlike general ITP prediction models which rely heavily on bleeding history or response to therapy (a retrospective criterion) (50–52), our model utilizes baseline parameters available at initial assessment. Compared to the CMQCC maternal hemorrhage risk stratification (53, 54) or general HIV prognosis scores (55–57), our nomogram is disease-specific, successfully disentangling the specific contribution of autoimmunity from the background noise of chronic HIV infection. By quantifying thrombocytopenia risk in the context of antibody status and immune reserves, the nomogram may help prioritize patients for further immunological workup, thereby supporting more informed clinical decision-making. As treatment response was not prospectively evaluated in this study, the therapeutic implications of these risk estimates require confirmation in prospective cohorts.

### 4.4 Model validation and robustness

The robustness of our model was confirmed through comprehensive internal validation. Bootstrap resampling (1,000 iterations) demonstrated minimal optimism (0.005) with bootstrap-corrected AUC of 0.857, and all iterations yielded AUC values above 0.78. Ten-fold cross-validation showed stable performance across folds (mean AUC 0.867 ± 0.055), confirming the absence of overfitting. Bootstrap coefficient stability analysis revealed that all four predictors maintained stable coefficients with 95% confidence intervals excluding zero. While GPIIIa showed greater variability (relative bias 29.6%) due to quasi-complete separation in approximately 5% of bootstrap samples—reflecting its exceptionally strong predictive value (94.2% thrombocytopenia rate among GPIIIa-positive patients)—the effect remained consistently positive across all 1,000 iterations, confirming robust predictive validity.

### 4.5 Limitations

Several limitations should be acknowledged. First, the cross-sectional design and relatively small sample size (N = 196) from a single designated regional HIV/AIDS treatment center — which may over-represent patients with more advanced disease stages relative to community-managed populations — limit generalizability; external validation in larger, multi-ethnic, prospective cohorts is warranted center. Second, while we infer hematopoietic suppression from peripheral blood indices, bone marrow biopsy data were unavailable to histologically confirm megakaryocyte status while. Third, our primary outcome was defined operationally rather than confirmed against an independent reference standard (e.g., treatment response, thrombopoietic markers, or adjudicated hematology diagnosis); the model therefore identifies an autoimmune-enriched phenotype rather than definitively confirmed ITP, and its utility for guiding specific immunomodulatory decisions requires prospective validation. Fourth, HIV viral load data were unavailable for a substantial proportion of patients, reflecting ART-naïve status at enrollment, receipt of ART at external facilities with inaccessible records, and non-universal testing intervals, precluding formal assessment of viral replication as an independent risk factor. While CD4+ T-cell count serves as an established surrogate for HIV-related immune dysregulation, it cannot fully substitute for direct viral load measurement, and future prospective studies incorporating routine viral load data are warranted. Fifth, although pre-specified sensitivity analyses (including adjustment for age, sex, ART status, and ART duration, as well as stratified modeling within ART-treated patients) demonstrated model stability, residual confounding by unmeasured ART-related factors (such as specific antiretroviral regimens, adherence patterns, treatment interruptions, and timing relative to thrombocytopenia onset) cannot be entirely excluded. Sixth, platelet-specific antibodies were measured qualitatively (positive/negative) rather than quantitatively; future studies incorporating antibody titers may reveal dose-response relationships between autoantibody levels and thrombocytopenia severity. Finally, the lack of longitudinal follow-up precluded assessment of how antibody status and CD4 trajectories jointly influence treatment response and platelet recovery over time.

## 5 Conclusion

In conclusion, we developed and internally validated a parsimonious 4-variable clinical prediction model that identifies an autoimmune-enriched thrombocytopenia phenotype in HIV-infected patients by integrating anti-GPIIIa antibody status with immunological and hematological reserves. The model demonstrated robust discriminative performance (AUC 0.862) and strong specificity for ruling in antibody-mediated disease, with stability confirmed across multiple pre-specified sensitivity analyses.

Our dose-response analysis revealed two clinically distinct risk trajectories stratified by antibody status: an antibody-negative group in which thrombocytopenia risk declined progressively with CD4 recovery, and an antibody-positive group that maintained markedly elevated risk across the observed CD4 range, suggesting that immune reconstitution alone may be insufficient to resolve thrombocytopenia in a subset of patients, though this observation requires confirmation in prospective studies.

The nomogram translates these findings into a bedside risk-stratification tool that may support clinical decision-making by identifying patients who warrant further immunological evaluation and targeted workup. However, as the primary outcome was defined operationally and treatment response was not prospectively evaluated, the model’s ability to guide specific immunomodulatory interventions requires validation in prospective cohorts before clinical implementation. External validation in larger, multi-ethnic populations is essential to confirm generalizability.

## Data Availability

The original contributions presented in the study are included in the article/**Supplementary Material**. Further inquiries can be directed to the corresponding author/s.
