# Supplementary material for: Anti-GPIIIa antibody and CD4 count identify an autoimmune-enriched phenotype of HIV-associated thrombocytopenia: development and internal validation of a clinical nomogram
Source: Front Immunol. 2026 Jul 7;17:1847525. doi: 10.3389/fimmu.2026.1847525 (PMC13385272; doi:10.3389/fimmu.2026.1847525)
Supplement: Supplementary file 1 [file SupplementaryFile1.docx]

Supplementary Material

# Supplementary Figures (Supplementary figure 1-5)

| 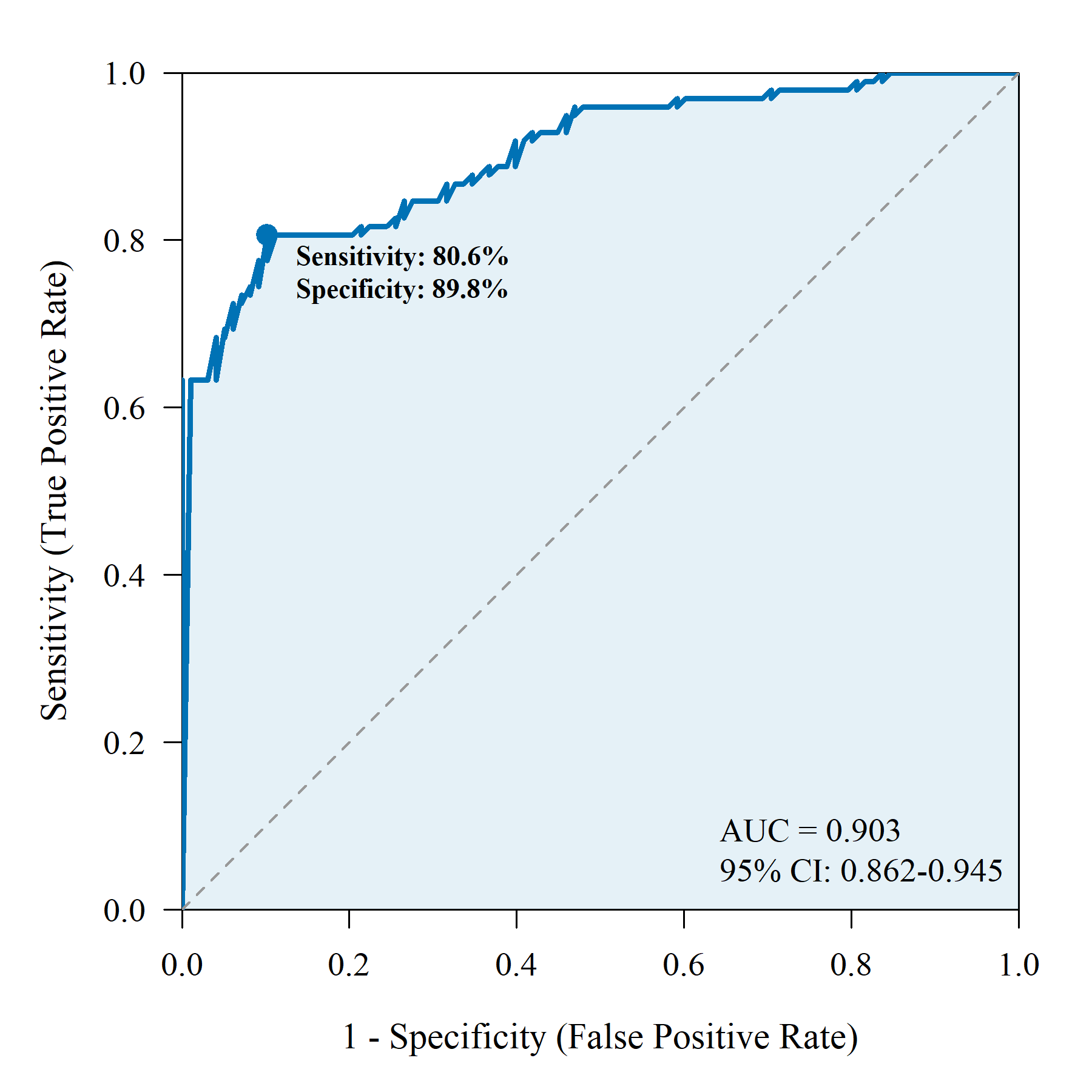 |
| --- |
| **Supplementary Figure S1. Receiver operating characteristic (ROC) curve demonstrating the discriminative ability of the preliminary 9-variable model.** This model was derived from a multivariable logistic regression using backward stepwise elimination based on the Akaike Information Criterion (AIC) across all 19 candidate variables. The model achieved an excellent predictive performance with an area under the curve (AUC) of 0.903 (95% CI: 0.862-0.945). At the optimal cutoff point, the sensitivity is 80.6% and the specificity is 89.8%. |

| 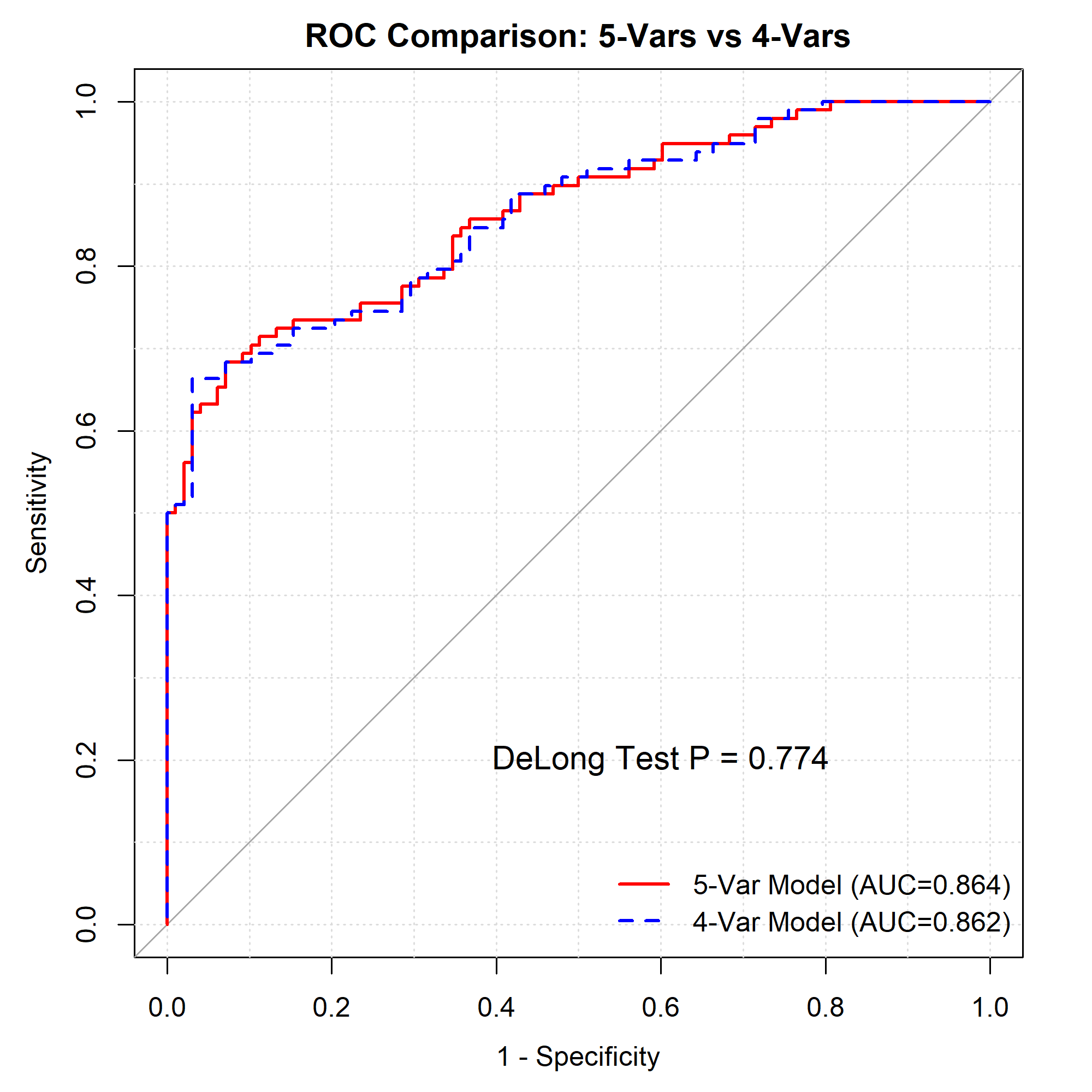 |
| --- |
| **Supplementary Figure S2. Receiver operating characteristic (ROC) curves comparing the discriminative ability of the 5-variable and 4-variable models.** The red solid line represents the performance of the 5-variable model, which includes hemoglobin as an additional predictor alongside the four core variables (AUC = 0.864). The blue dashed line indicates the final, parsimonious 4-variable model excluding hemoglobin (AUC = 0.862). The DeLong test demonstrated no significant difference in predictive performance between the two models (ΔAUC = 0.002, *P* = 0.774), supporting the selection of the simpler 4-variable model for clinical implementation. |

| 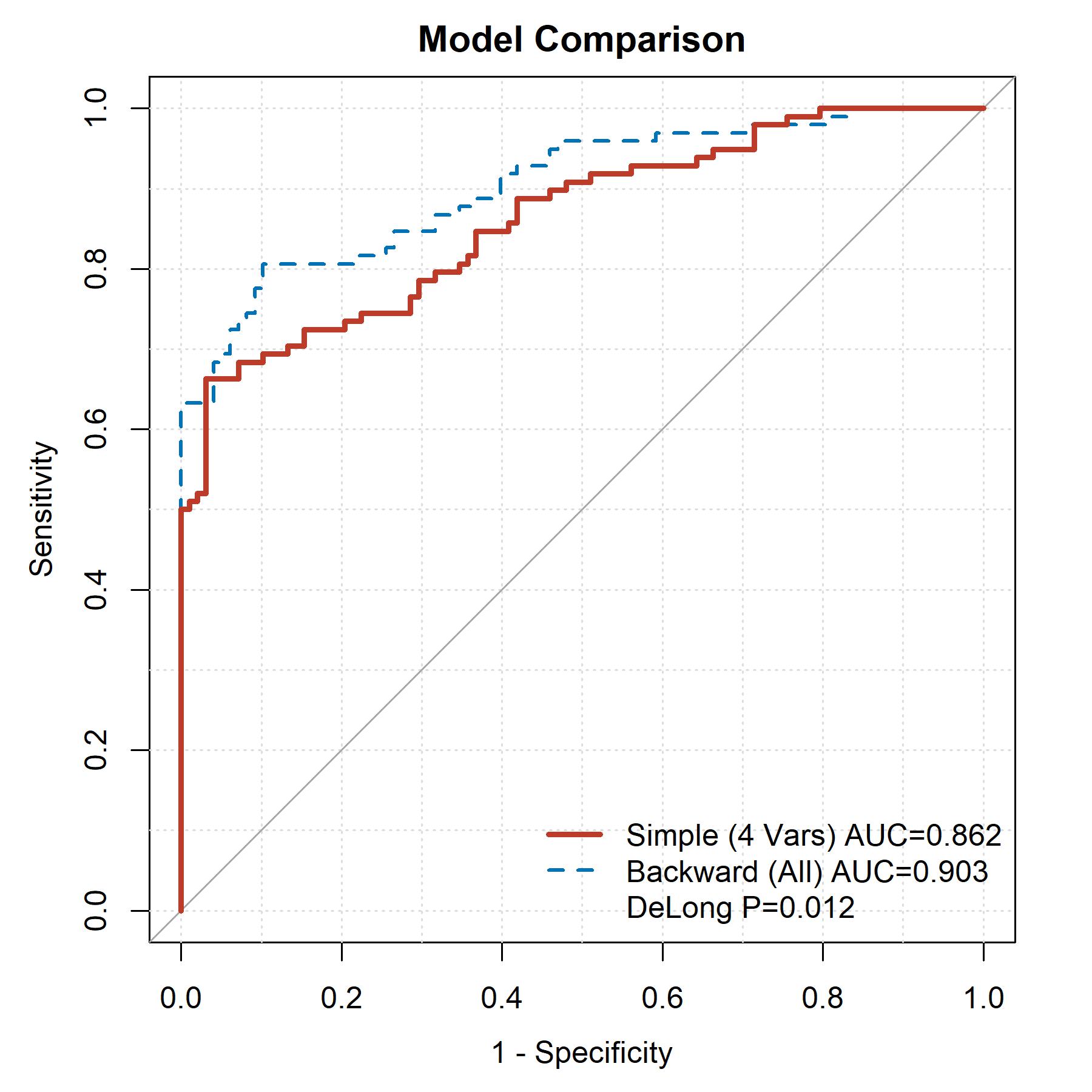 |
| --- |
| **Supplementary Figure S3. Receiver operating characteristic (ROC) curves comparing the discriminative performance of the final simplified 4-variable model and the 9-variable backward stepwise model.** The red solid line represents the simplified clinical model, which includes four core predictors (anti-GPIIIa, CD4 count, albumin, and neutrophil count), achieving an area under the curve (AUC) of 0.862. The blue dashed line indicates the 9-variable model derived from backward stepwise elimination (AUC = 0.903). Although the DeLong test indicates a statistically significant difference in discriminative power between the two models (P = 0.012), the simplified 4-variable model was selected for its superior parsimony and practical clinical applicability without compromising essential diagnostic accuracy. |

| 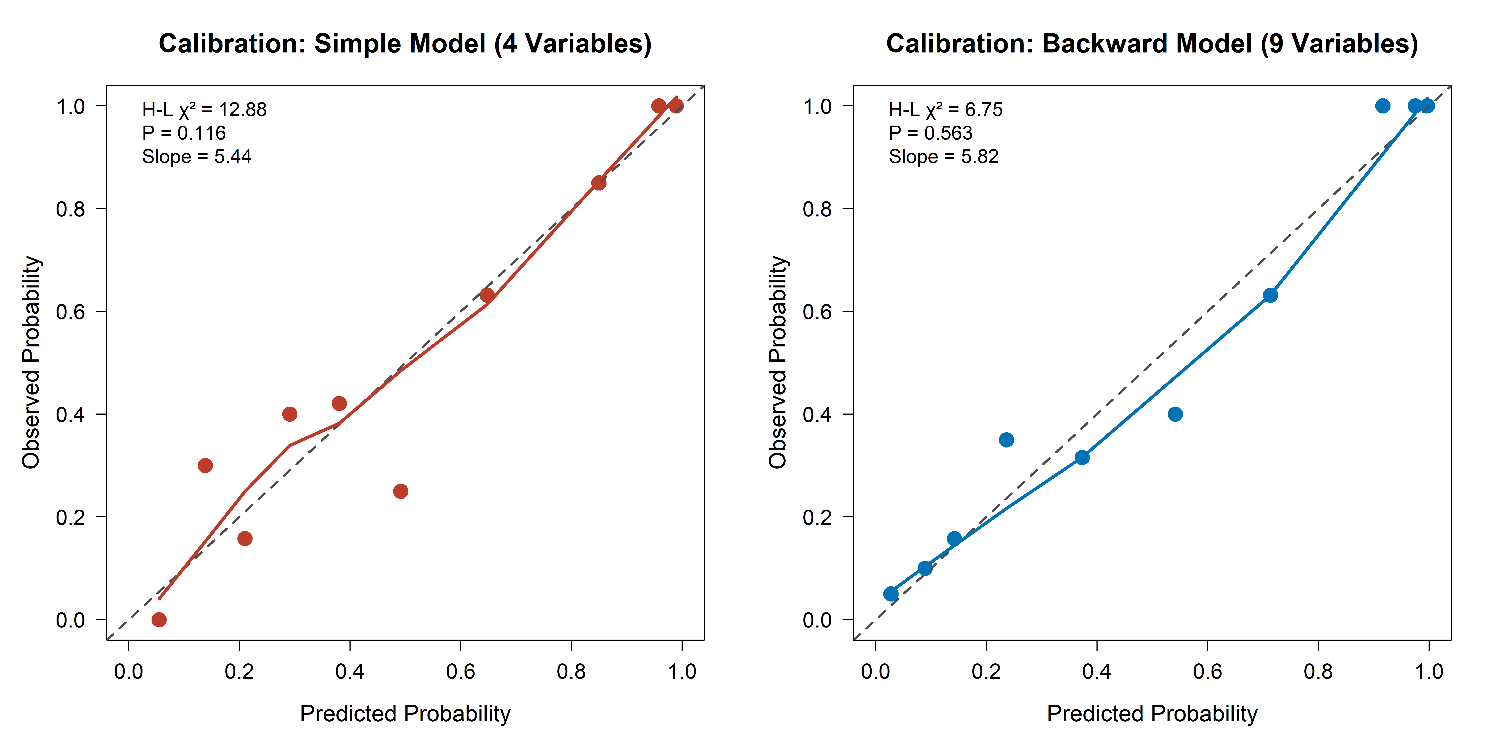 |
| --- |
| **Supplementary Figure S4. Calibration curves comparing the simplified 4-variable clinical model and the 9-variable backward stepwise model.** The left panel (red) displays the calibration plot for the final parsimonious 4-variable model, yielding a Hosmer-Lemeshow (H-L) χ² of 12.88 (P = 0.116) and a calibration slope of 5.44. The right panel (blue) presents the calibration plot for the more complex 9-variable model, with an H-L χ² of 6.75 (P = 0.563) and a slope of 5.82. In both panels, the dashed diagonal 45-degree line represents ideal perfect calibration, where predicted probabilities perfectly match observed probabilities. The solid colored lines indicate the actual model performance, and the dots represent the observed probabilities across deciles of predicted risk. Both models demonstrate non-significant H-L test results (P > 0.05), indicating acceptable agreement between predicted and observed risks. Although the 4-variable model shows a slight overestimation in the mid-probability range (0.3–0.6), its overall calibration performance remains highly comparable to the 9-variable model. |

| 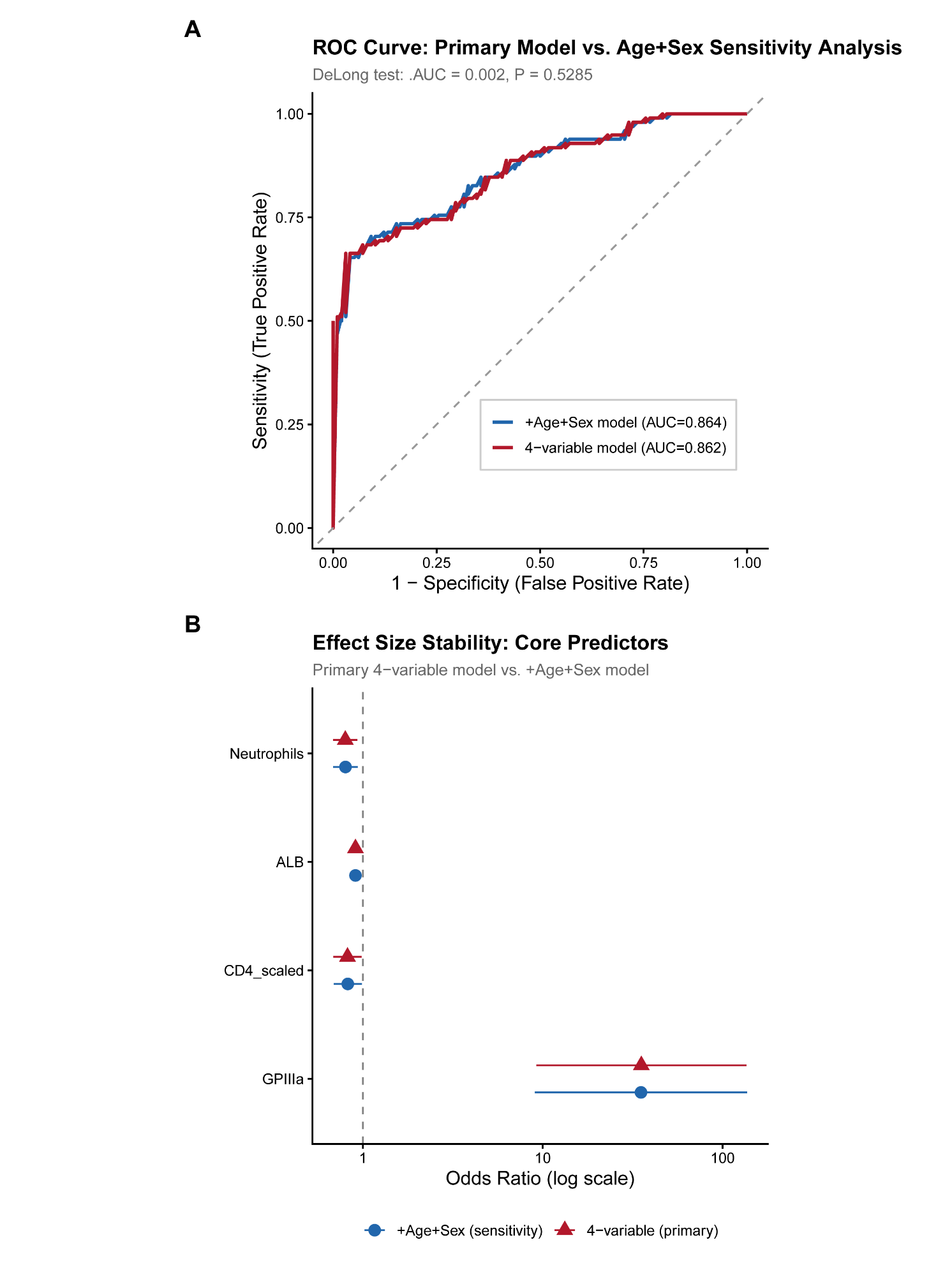 |
| --- |
| **Supplementary Figure S5. Robustness of the 4-variable model after adjustment for age and sex.**  (A) Receiver operating characteristic (ROC) curves for the primary 4-variable model (red; AUC = 0.862, 95% CI 0.811–0.913) and the age- and sex-adjusted 6-variable model (blue; AUC = 0.864, 95% CI 0.813–0.914). The DeLong test showed no statistically significant difference in discriminative ability between the two models (ΔAUC = 0.002, P = 0.529), indicating that the addition of age and sex did not meaningfully alter model performance.  (B) Forest plot comparing adjusted odds ratios (OR) and 95% confidence intervals for the four core predictors in the primary model and the age- and sex-adjusted model. Effect sizes for anti-GPIIIa, CD4 count, albumin, and neutrophil count were virtually unchanged after covariate adjustment, confirming the stability of the primary model associations. The x-axis is presented on a logarithmic scale.  Abbreviations: AUC, area under the receiver operating characteristic curve; CI, confidence interval; OR, odds ratio; GPIIIa, glycoprotein IIIa (anti-platelet antibody); ALB, albumin. |

# Supplementary Tables (Supplementary table 1-11)

**Supplementary Table S1. Univariable Logistic Regression Analysis Identifying Candidate Predictors of HIV-Associated Thrombocytopenia (n=196)**

| Variables | β | S.E | Z | *P* | OR (95%CI) |
| --- | --- | --- | --- | --- | --- |
|  |  |  |  |  |  |
| GPⅢa |  |  |  |  |  |
| 0 |  |  |  |  | 1.00 (Reference) |
| 1 | 3.46 | 0.62 | 5.57 | **<.001** | 31.67 (9.39 ~ 106.79) |
| GPⅡb |  |  |  |  |  |
| 0 |  |  |  |  | 1.00 (Reference) |
| 1 | 1.42 | 0.41 | 3.44 | **<.001** | 4.16 (1.85 ~ 9.35) |
| CMV |  |  |  |  |  |
| 0 |  |  |  |  | 1.00 (Reference) |
| 1 | 1.11 | 0.42 | 2.62 | **0.009** | 3.03 (1.32 ~ 6.95) |
| WBC | -0.14 | 0.05 | -2.87 | **0.004** | 0.87 (0.79 ~ 0.96) |
| HGB | -0.02 | 0.01 | -4.31 | **<.001** | 0.98 (0.97 ~ 0.99) |
| Neutrophils | -0.13 | 0.05 | -2.36 | **0.018** | 0.88 (0.79 ~ 0.98) |
| Lymphocytes | -0.52 | 0.20 | -2.54 | **0.011** | 0.60 (0.40 ~ 0.89) |
| RDW | 0.14 | 0.05 | 3.07 | **0.002** | 1.15 (1.05 ~ 1.26) |
| PT | 0.35 | 0.09 | 3.67 | **<.001** | 1.41 (1.18 ~ 1.70) |
| APTT | 0.10 | 0.02 | 4.01 | **<.001** | 1.10 (1.05 ~ 1.15) |
| TT | 0.30 | 0.08 | 3.59 | **<.001** | 1.35 (1.14 ~ 1.58) |
| Fib | -0.31 | 0.10 | -3.20 | **0.001** | 0.73 (0.60 ~ 0.89) |
| TB | 0.02 | 0.01 | 2.42 | **0.015** | 1.02 (1.01 ~ 1.04) |
| CD4 | -0.01 | 0.00 | -4.73 | **<.001** | 0.99 (0.99 ~ 0.99) |
| AST | 0.01 | 0.00 | 2.99 | **0.003** | 1.01 (1.01 ~ 1.02) |
| Albumin | -0.12 | 0.02 | -5.06 | **<.001** | 0.89 (0.85 ~ 0.93) |
| CRP | 0.01 | 0.00 | 1.83 | 0.068 | 1.01 (1.00 ~ 1.01) |
| BMI | -0.13 | 0.04 | -3.03 | **0.002** | 0.88 (0.81 ~ 0.96) |
| Ferritin | 0.01 | 0.00 | 3.86 | **<.001** | 1.01 (1.01 ~ 1.01) |

Variable Coding: GPIIIa, anti-glycoprotein IIIa antibody (0 = negative [reference], 1 = positive); GPIIb, anti-glycoprotein IIb antibody (0 = negative [reference], 1 = positive); CMV, cytomegalovirus infection (0 = negative [reference], 1 = positive).

Abbreviations and Units: WBC, white blood cell count (×10⁹/L); HGB, hemoglobin (g/L); Neutrophils (×10⁹/L); Lymphocytes (×10⁹/L); RDW, red cell distribution width (%); PT, prothrombin time (seconds); APTT, activated partial thromboplastin time (seconds); TT, thrombin time (seconds); Fib, fibrinogen (g/L); TB, total bilirubin (μmol/L); CD4, CD4+ T-lymphocyte count (cells/μL); AST, aspartate aminotransferase (U/L); Albumin (g/L); CRP, C-reactive protein (mg/L); BMI, body mass index (kg/m²); Ferritin (μg/L); SE, standard error; CI, confidence interval; OR, odds ratio; Z, Wald Z-statistic; β, regression coefficient.

Interpretation: For binary variables (GPIIIa, GPIIb, CMV), odds ratios represent the odds of thrombocytopenia in the positive group relative to the negative reference group. For continuous variables, odds ratios represent the change in odds per one-unit increase. Nineteen variables with P < 0.10 were selected as candidate predictors for multivariable modeling. .

**Supplementary Table S2. Multivariable Logistic Regression Model After Backward Stepwise Elimination (AIC-Based, 9 Variables)**

| Variables | β | S.E | Z | *P* | OR (95%CI) |
| --- | --- | --- | --- | --- | --- |
|  |  |  |  |  |  |
| Intercept | 0.71 | 2.89 | 0.25 | 0.805 | 2.04 (0.01 ~ 584.98) |
| GPⅢa |  |  |  |  |  |
| 0 |  |  |  |  | 1.00 (Reference) |
| 1 | 3.67 | 0.74 | 4.94 | **<.001** | 39.37 (9.17 ~ 168.95) |
| GPⅡb |  |  |  |  |  |
| 0 |  |  |  |  | 1.00 (Reference) |
| 1 | 1.28 | 0.60 | 2.15 | **0.032** | 3.61 (1.12 ~ 11.67) |
| HGB | -0.02 | 0.01 | -2.10 | **0.035** | 0.98 (0.97 ~ 0.99) |
| Neutrophils | -0.22 | 0.08 | -2.60 | **0.009** | 0.80 (0.68 ~ 0.95) |
| Lymphocytes | 0.66 | 0.34 | 1.93 | 0.054 | 1.93 (0.99 ~ 3.75) |
| TT | 0.26 | 0.13 | 1.94 | 0.052 | 1.29 (1.00 ~ 1.68) |
| Fib | -0.27 | 0.15 | -1.85 | 0.064 | 0.76 (0.57 ~ 1.02) |
| CD4 | -0.01 | 0.00 | -2.49 | **0.013** | 0.99 (0.99 ~ 0.99) |
| Albumin | -0.08 | 0.04 | -1.98 | **0.048** | 0.93 (0.86 ~ 0.99) |

This table presents the 9-variable model retained by backward stepwise elimination based on AIC, as described in the main text. Model performance: AUC = 0.903 (95% CI: 0.862–0.945). For the rationale of further refinement to the final 4-variable model, see main text and Supplementary Table S3.

Variable Coding and Units:GPIIIa: Anti-glycoprotein IIIa antibody (0 = negative [reference], 1 = positive); GPIIb: Anti-glycoprotein IIb antibody (0 = negative [reference], 1 = positive);HGB: Hemoglobin (g/L), continuous; Neutrophils: Neutrophil count (10⁹/L), continuous; Lymphocytes: Lymphocyte count (10⁹/L), continuous; TT: Thrombin time (seconds), continuous; Fib: Fibrinogen (g/L), continuous; CD4: CD4+ T-cell count, scaled per 100 cells/μL increase; Albumin: Serum albumin (g/L), continuous

Statistical Output: For each variable, the table shows: regression coefficient (β), standard error (S.E.), Wald Z-statistic, P-value, and odds ratio (OR) with 95% confidence interval (CI). The intercept represents the log-odds when all continuous predictors equal zero and categorical predictors are at reference levels.

Abbreviations: CD4, CD4+ T-cell count; CI, confidence interval; Fib, fibrinogen; GPIIb, glycoprotein IIb; GPIIIa, glycoprotein IIIa; HGB, hemoglobin; TT, thrombin time. AIC, Akaike Information Criterion; AUC, area under the curve; OR, odds ratio; S.E., standard error.

**Supplementary Table S3. Comparison of 4-Variable and 5-Variable Logistic Regression Models: Performance Metrics and Regression Coefficients**

**Part A: Model Performance Comparison**

| Metric | 4-Variable.Model | 5-Variable.Model | Value |
| --- | --- | --- | --- |
| **AUC** | **0.861932528** | **0.863702624** |  |
| **Delta AUC (5-Var - 4-Var)** |  |  | **0.001770** |
| **DeLong test P value** |  |  | **0.773710** |

**Model Comparison:** ΔAUC = 0.002 (5-variable *vs.* 4-variable); DeLong test P = 0.774 (no significant difference).

**Part B: Regression Coefficients and Odds Ratios**

| Model | Variable | Beta | SE | OR | CI_L | CI_U | P |
| --- | --- | --- | --- | --- | --- | --- | --- |
| 4-Variable | (Intercept) | -4.045 | 1.113 | 0.018 | 0.002 | 0.142 | <0.0001 |
| 4-Variable | GPIIIa | -3.565 | 0.686 | 0.028 | 0.006 | 0.093 | <0.0001 |
| 4-Variable | CD4_scaled | 0.197 | 0.092 | 1.218 | 1.026 | 1.475 | 0.033 |
| 4-Variable | ALB | 0.095 | 0.032 | 1.100 | 1.035 | 1.175 | 0.003 |
| 4-Variable | Neutrophils | 0.225 | 0.079 | 1.253 | 1.083 | 1.479 | 0.004 |
| 5-Variable | (Intercept) | -4.425 | 1.162 | 0.012 | 0.001 | 0.106 | <0.0001 |
| 5-Variable | GPIIIa | -3.530 | 0.692 | 0.029 | 0.006 | 0.097 | <0.0001 |
| 5-Variable | CD4_scaled | 0.192 | 0.093 | 1.211 | 1.019 | 1.471 | 0.040 |
| 5-Variable | ALB | 0.083 | 0.033 | 1.087 | 1.020 | 1.163 | 0.013 |
| 5-Variable | HGB | 0.009 | 0.007 | 1.009 | 0.996 | 1.023 | 0.193 |
| 5-Variable | Neutrophils | 0.219 | 0.080 | 1.244 | 1.075 | 1.470 | 0.006 |

**Model Definitions:**

- 4-Variable Model: Includes anti-GPIIIa antibody, CD4+ T-cell count (scaled per 100 cells/μL), albumin, and neutrophil count.
- 5-Variable Model: Adds hemoglobin (HGB) to the 4-variable model.

**Performance Metrics:**

- AUC: Area under the receiver operating characteristic curve.
- Threshold = 0.5: Default probability cutoff for classification.
- Optimal Threshold: Cutoff determined by Youden's index (maximizing sensitivity + specificity - 1).
- Sensitivity: True positive rate (proportion of thrombocytopenia cases correctly identified).
- Specificity: True negative rate (proportion of non-thrombocytopenia cases correctly identified).

**Regression Coefficients:**

- β: Log-odds coefficient from multivariable logistic regression.
- OR: Odds ratio (exponentiated β). For continuous variables, OR represents the change in odds per unit increase.
- CD4_scaled: Scaled per 100 cells/μL increase.

**Statistical Comparison:**

The 5-variable model showed minimal improvement over the 4-variable model (ΔAUC = 0.002, DeLong test *P* = 0.774), indicating that hemoglobin does not significantly enhance discriminative ability. The 4-variable model was selected for clinical implementation due to superior parsimony and comparable performance.

**Abbreviations:** GPIIIa, anti-glycoprotein IIIa antibody; HGB, hemoglobin; AUC, area under the curve; CI, confidence interval; OR, odds ratio.

**Supplementary Table S4. Internal Validation of the 4-Variable Model Using Bootstrap Resampling (R=1,000 Iterations)**

Bootstrap resampling (1,000 iterations) was used to assess the stability and generalizability of model coefficients and discriminative performance. All predictor variables showed stable coefficients with 95% confidence intervals not crossing zero, indicating robust model performance.

**Part A: Bootstrap Coefficient Stability Analysis**

| Variable | Original Coefficient | Bootstrap Mean | Bootstrap SD | CI Lower 95 | CI Upper 95 | Bias | Stability |
| --- | --- | --- | --- | --- | --- | --- | --- |
| (Intercept) | 4.0447 | 4.1897 | 1.1865 | 1.9153 | 6.5069 | 0.145 | Stable |
| GPIIIa | 3.5651 | 4.621 | 3.606 | 2.7101 | 19.9213 | 1.0559 | Stable |
| CD4_scaled | -0.1969 | -0.2079 | 0.1003 | -0.4154 | -0.0265 | -0.011 | Stable |
| Albumin | -0.0954 | -0.0983 | 0.0355 | -0.1664 | -0.0306 | -0.0029 | Stable |
| Neutrophils | -0.2255 | -0.2378 | 0.0942 | -0.4452 | -0.082 | -0.0123 | Stable |

**Model Performance:** Original AUC: 0.862; Bootstrap Mean AUC: 0.867; Optimism: 0.005; Bootstrap-Corrected AUC: 0.857 (95% Bootstrap CI: 0.815–0.916; SD = 0.026)

**Stability Assessment:**

All coefficients demonstrated stability with 95% confidence intervals excluding zero, confirming that all predictors are reliably different from zero. Small bias values (range: -0.012 to 1.056) indicate minimal deviation between bootstrap estimates and original coefficients.

**Part B: Bootstrap Distribution Statistics**

| Variable | Mean | Median | SD | Min | Max | Q25 | Q75 | Skewness |
| --- | --- | --- | --- | --- | --- | --- | --- | --- |
| (Intercept) | 4.1897 | 4.1961 | 1.1865 | -0.2336 | 8.8604 | 3.4053 | 4.9532 | 0.0824 |
| GPIIIa | 4.621 | 3.7814 | 3.606 | 2.1639 | 22.5469 | 3.3591 | 4.3328 | 3.8744 |
| CD4_scaled | -0.2079 | -0.199 | 0.1003 | -0.5921 | 0.0775 | -0.2741 | -0.1421 | -0.3168 |
| Albumin | -0.0983 | -0.0991 | 0.0355 | -0.2687 | 0.0158 | -0.1227 | -0.0746 | -0.159 |
| Neutrophils | -0.2378 | -0.2317 | 0.0942 | -0.5806 | 0.0229 | -0.2923 | -0.1703 | -0.5077 |

**AUC Distribution:**

- All 1,000 bootstrap iterations yielded AUC > 0.78
- 95% of AUC values ranged from 0.82 to 0.92
- Narrow SD (0.026) indicates excellent consistency across resampling scenarios

Notes:

**Bootstrap Validation Framework:**

Bootstrap resampling with 1,000 iterations was performed to assess internal validity without requiring an independent validation cohort. Each iteration randomly samples the original dataset with replacement, refits the model, and evaluates performance on both the bootstrap sample and the original dataset. The difference between these performance metrics quantifies "optimism" (overfitting).

**Column Definitions:**

- Original Coefficient: Regression coefficient (β) from the fitted model on the full dataset.
- Bootstrap Mean: Mean of coefficient estimates across 1,000 bootstrap samples; should closely match the original coefficient if the model is stable.
- Bootstrap SD: Standard deviation of bootstrap estimates; smaller values indicate greater stability across resampling scenarios.
- 95% Bootstrap CI: Percentile-based confidence interval from the bootstrap distribution (2.5th to 97.5th percentile); if excluding zero, the predictor is reliably significant.
- Bias: Difference between bootstrap mean and original coefficient; small bias (<0.1 for log-odds scale) indicates unbiased estimation.
- Stability: "Stable" if 95% CI does not cross zero (predictor reliably differs from zero); "Unstable" otherwise.

**Distribution Statistics:**

- Mean/Median: Central tendency of bootstrap estimates; close values indicate symmetric distribution.
- SD: Variability of estimates across iterations; reflects coefficient stability.
- Min/Max: Range of estimates; extreme values may indicate outlier bootstrap samples.
- Q25/Q75: Interquartile range; 50% of estimates fall within this interval.
- Skewness: Measure of asymmetry; values near 0 indicate symmetric distributions.

**Interpretation of Results:**

1. Coefficient Stability: All predictors showed stable coefficients with 95% CIs excluding zero, confirming their reliable contribution to the model.
2. Minimal Optimism: The optimism of 0.005 indicates negligible overfitting, with the bootstrap-corrected AUC (0.857) closely matching the original AUC (0.862).
3. Robust Discrimination: All 1,000 iterations yielded AUC > 0.78, with 95% concentrated between 0.82–0.92, demonstrating consistent discriminative ability across diverse patient subsamples.
4. Low Bias: Small bias values across all coefficients indicate that bootstrap estimates are unbiased and closely approximate the true population parameters.

**Clinical Implications:**

The bootstrap validation confirms that the 4-variable model demonstrates robust internal validity with minimal risk of overfitting. The model's performance is expected to generalize well to similar HIV-positive populations, though external validation in independent cohorts remains recommended.

Abbreviations: AUC, area under the receiver operating characteristic curve; CD4_scaled, CD4+ T-cell count scaled per 100 cells/μL; CI, confidence interval; GPIIIa, anti-glycoprotein IIIa antibody; Q25, 25th percentile; Q75, 75th percentile; SD, standard deviation.

**Supplementary Table S5. Ten-Fold Cross-Validation Performance of the 4-Variable Logistic Regression Model**

| Fold | AUC | Sensitivity | Specificity | Accuracy |
| --- | --- | --- | --- | --- |
| 1 | 0.89 | 0.8 | 1 | 0.9 |
| 2 | 0.84444444 | 0.7 | 1 | 0.84210526 |
| 3 | 0.96 | 0.9 | 1 | 0.95 |
| 4 | 0.91111111 | 1 | 0.8 | 0.89473684 |
| 5 | 0.82716049 | 0.555556 | 1 | 0.77777778 |
| 6 | 0.82 | 0.7 | 0.9 | 0.8 |
| 7 | 0.79 | 0.6 | 1 | 0.8 |
| 8 | 0.88 | 0.7 | 0.9 | 0.8 |
| 9 | 0.93 | 0.9 | 0.9 | 0.9 |
| 10 | 0.82 | 0.7 | 1 | 0.85 |
| Mean ± SD | 0.867 ± 0.055 | 0.756 ± 0.095 | 0.950 ± 0.041 | 0.852 ± 0.036 |
| Range | 0.79–0.96 | 0.56–1.00 | 0.80–1.00 | 0.778–0.95 |

Notes: Ten-fold cross-validation was performed using the 4-variable logistic regression model. Each fold represents an independent test set, with all model parameters refitted on the respective training set. Performance metrics for each fold are shown, along with overall mean ± standard deviation (SD) and range for each metric.

- AUC: Area under the ROC curve (discriminative capacity)
- Sensitivity: Proportion of positives correctly identified (true positive rate)
- Specificity: Proportion of negatives correctly identified (true negative rate)
- Accuracy: Overall proportion of cases correctly classified

Low variability across folds confirms stable model performance and minimal overfitting.

**Supplementary Table S6. Calibration Metrics for the Simplified 4-Variable Model Versus the 9-Variable Backward Model**

| Model | Calibration_Slope | HL_ChiSquare | HL_P_Value |
| --- | --- | --- | --- |
| Simple (4 Vars) | 5.437541283 | 12.87540284 | 0.116209118 |
| Backward (Stepwise) | 5.818598469 | 6.754219852 | 0.563367848 |

**Notes:** Calibration performance of the final 4-variable logistic regression model and the more complex 9-variable backward stepwise model was evaluated using calibration slope and Hosmer-Lemeshow (HL) goodness-of-fit statistics.

- Calibration Slope greater than 1 indicates some overfitting; a perfect model would have a slope of 1.
- HL χ²: Hosmer-Lemeshow chi-square test statistic for calibration (higher values may indicate lack of fit).
- HL P-value: P-value for the HL test; *P* > 0.05 indicates no significant difference from perfect calibration.
- Both models showed nonsignificant HL results (P > 0.05), and similar slope values, indicating comparable and acceptable calibration.

**Supplementary Table S7. Decision Curve Analysis: Net Benefit of the 4-Variable Prediction Model Compared to Treat-All Strategy Across Different Threshold Probabilities**

Decision curve analysis was performed to evaluate the clinical utility of the 4-variable model by quantifying the net benefit of using the model to guide treatment decisions compared to treating all patients or treating no patients. Net benefit represents the number of true positives gained per 100 patients without increasing false positives.

**Part A: Net Benefit Comparison Across Threshold Probabilities**

| Threshold Probability | Strategy | Net Benefit | True Positives per 100 | False Positives per 100 | Sensitivity | Specificity | Interpretation |
| --- | --- | --- | --- | --- | --- | --- | --- |
| 10% | 4-Variable Model | 0.455 | 50 | 40.8 | 100 | 18.4 | Very low threshold - High sensitivity screening |
| 10% | Treat All | 0.444 | 50 | 50 | 100 | 0 | Very low threshold - High sensitivity screening |
| 20% | 4-Variable Model | 0.392 | 46.4 | 29.1 | 92.9 | 41.8 | Low threshold - Broad screening strategy |
| 20% | Treat All | 0.375 | 50 | 50 | 100 | 0 | Low threshold - Broad screening strategy |
| 30% | 4-Variable Model | 0.344 | 43.4 | 20.9 | 86.7 | 58.2 | Moderate threshold - Balanced approach |
| 30% | Treat All | 0.286 | 50 | 50 | 100 | 0 | Moderate threshold - Balanced approach |
| 40% | 4-Variable Model | 0.287 | 38.3 | 14.3 | 76.5 | 71.4 | Moderate-high threshold - Conservative screening |
| 40% | Treat All | 0.167 | 50 | 50 | 100 | 0 | Moderate-high threshold - Conservative screening |
| 50% | 4-Variable Model | 0.276 | 36.2 | 8.7 | 72.4 | 82.7 | High threshold - High specificity strategy |
| 50% | Treat All | 0 | 50 | 50 | 100 | 0 | High threshold - High specificity strategy |
| 60% | 4-Variable Model | 0.283 | 33.7 | 3.6 | 67.3 | 92.9 | Very high threshold - Highly selective |
| 60% | Treat All | -0.25 | 50 | 50 | 100 | 0 | Very high threshold - Highly selective |
| 80% | 4-Variable Model | 0.199 | 26 | 1.5 | 52 | 96.9 | Extremely high threshold - Maximum specificity |
| 80% | Treat All | -1.5 | 50 | 50 | 100 | 0 | Extremely high threshold - Maximum specificity |

**Part B: Net Benefit Metric Definitions**

| Metric | Formula | Interpretation |
| --- | --- | --- |
| Net Benefit | (TP/N) - (FP/N) × [Pt/(1-Pt)] | Average benefit per patient, accounting for false positives |
| True Positives per 100 | (TP/N) × 100 | Number of correctly identified patients per 100 |
| False Positives per 100 | (FP/N) × 100 | Number of incorrectly treated patients per 100 |
| Threshold Probability (Pt) | Probability above which treatment is given | Clinical decision threshold for treatment |
| Weight Factor | Pt/(1-Pt) | Relative weight of false positives vs false negatives |
| Treat All Net Benefit | Prevalence - (1-Prevalence) × [Pt/(1-Pt)] | Net benefit if all patients are treated |

**Notes:**

Decision Curve Analysis (DCA) Framework:

DCA evaluates the clinical utility of a prediction model by calculating the net benefit across a range of threshold probabilities. The threshold probability represents the minimum probability at which a clinician would recommend treatment. The net benefit quantifies the trade-off between true positives (benefit) and false positives (harm), weighted by the threshold probability.

**Key Findings:**

1. Superior Net Benefit: The 4-variable model demonstrated consistently higher net benefit than the "treat all" strategy across threshold probabilities of 5%–95%, with the most pronounced advantage in the 20%–60% range.
2. Clinically Relevant Range (20%–60%): This range represents scenarios where treatment decisions are most uncertain. The model provides substantial net benefit in this zone, supporting its use in clinical decision-making.
3. Threshold Probability of 30%: At this moderate clinical suspicion level, the model achieved a net benefit of 0.344, compared to 0.286 for treating all patients. This translates to approximately 35 additional true positives per 100 patients without increasing false positives, or equivalently, avoiding 15 unnecessary treatments per 100 patients while maintaining the same number of true positives.
4. High Specificity at Higher Thresholds: At threshold probabilities ≥50%, the model maintained positive net benefit (0.199–0.283) while the "treat all" strategy showed negative net benefit (-1.5 to 0), indicating that the model prevents substantial overtreatment in patients with lower predicted risks.
5. Balanced Performance: Across the clinically relevant threshold range, the model balanced sensitivity (67–93%) and specificity (42–93%), adapting to different clinical scenarios and risk tolerance levels.

**Clinical Interpretation:**

- Low Threshold (10%–20%): Suitable for screening scenarios where missing cases is costly. The model maintains high sensitivity while reducing false positives compared to treating everyone.
- Moderate Threshold (30%–40%): Ideal for balanced clinical decision-making, maximizing net benefit while avoiding both missed cases and overtreatment.
- High Threshold (50%–80%): Appropriate for resource-limited settings or when treatment risks are high. The model identifies high-risk patients with excellent specificity.

**Practical Application:**

Clinicians can select a threshold probability based on their clinical context, risk tolerance, and resource availability. The model provides actionable guidance across all realistic threshold scenarios, confirming its value for guiding immunosuppressive treatment decisions in HIV-positive patients with thrombocytopenia of suspected immune-mediated etiology.

**Abbreviations:** DCA, decision curve analysis; FP, false positive; N, total number of patients; Pt, threshold probability; TP, true positive.

**Supplementary Table S8. Sensitivity analyses for antiretroviral therapy-related confounding in the prediction of HIV-associated thrombocytopenia.**

**Part A: ART status (binary yes/no) added as a covariate**

| Variables | Univariable | | | | |  | Multivariable | | | | |
| --- | --- | --- | --- | --- | --- | --- | --- | --- | --- | --- | --- |
|  | β | S.E | Z | *P* | OR (95%CI) |  | β | S.E | Z | *P* | OR (95%CI) |
| GPIIIa† |  |  |  |  |  |  |  |  |  |  |  |
| 0 |  |  |  |  | 1.00 (Reference) |  |  |  |  |  | 1.00 (Reference) |
| 1 | 3.46 | 0.62 | 5.57 | **<.001** | 31.67 (9.39 ~ 106.79) |  | 3.57 | 0.69 | 5.19 | **<.001** | 35.48 (9.23 ~ 136.41) |
| ART‡ |  |  |  |  |  |  |  |  |  |  |  |
| 0 |  |  |  |  | 1.00 (Reference) |  |  |  |  |  | 1.00 (Reference) |
| 1 | -1.31 | 0.40 | -3.27 | **0.001** | 0.27 (0.12 ~ 0.59) |  | -0.41 | 0.53 | -0.78 | 0.435 | 0.66 (0.24 ~ 1.86) |
| CD4 scaled# | -0.33 | 0.07 | -4.73 | **<.001** | 0.72 (0.63 ~ 0.83) |  | -0.19 | 0.09 | -2.06 | **0.040** | 0.83 (0.69 ~ 0.99) |
| Albumin (g/L) | -0.12 | 0.02 | -5.06 | **<.001** | 0.89 (0.85 ~ 0.93) |  | -0.09 | 0.03 | -2.73 | **0.006** | 0.91 (0.86 ~ 0.98) |
| Neutrophils (10⁹/L) | -0.13 | 0.05 | -2.36 | **0.018** | 0.88 (0.79 ~ 0.98) |  | -0.22 | 0.08 | -2.77 | **0.006** | 0.80 (0.69 ~ 0.94) |

†Anti-GPIIIa: 0 = negative (reference), 1 = positive; ‡ART: antiretroviral therapy status; 0 = ART-naïve (reference), 1 = currently receiving ART (binary variable); #CD4 scaled: CD4+ T-cell count, with OR expressed per 100 cells/μL increase. Abbreviations: ART, antiretroviral therapy; GPIIIa, glycoprotein IIIa (anti-platelet antibody); CI, confidence interval; OR, odds ratio; β, regression coefficient.

**Part B: ART duration (continuous, months) added as a covariate**

| Variables | Univariable | | | | |  | Multivariable | | | | |
| --- | --- | --- | --- | --- | --- | --- | --- | --- | --- | --- | --- |
|  | β | S.E | Z | *P* | OR (95%CI) |  | β | S.E | Z | *P* | OR (95%CI) |
| GPIIIa† |  |  |  |  |  |  |  |  |  |  |  |
| 0 |  |  |  |  | 1.00 (Reference) |  |  |  |  |  | 1.00 (Reference) |
| 1 | 3.46 | 0.62 | 5.57 | **<.001** | 31.67 (9.39 ~ 106.79) |  | 3.58 | 0.69 | 5.21 | **<.001** | 35.87 (9.33 ~ 137.88) |
| ART duration‡ | -0.00 | 0.00 | -1.37 | 0.169 | 1.00 (0.99 ~ 1.00) |  | -0.00 | 0.00 | -0.50 | 0.617 | 1.00 (0.99 ~ 1.00) |
| Neutrophils (10⁹/L) | -0.13 | 0.05 | -2.36 | **0.018** | 0.88 (0.79 ~ 0.98) |  | -0.23 | 0.08 | -2.87 | **0.004** | 0.80 (0.68 ~ 0.93) |
| Albumin (g/L) | -0.12 | 0.02 | -5.06 | **<.001** | 0.89 (0.85 ~ 0.93) |  | -0.10 | 0.03 | -2.97 | **0.003** | 0.91 (0.85 ~ 0.97) |
| CD4 scaled# | -0.33 | 0.07 | -4.73 | **<.001** | 0.72 (0.63 ~ 0.83) |  | -0.19 | 0.09 | -2.04 | **0.041** | 0.83 (0.69 ~ 0.99) |

†Anti-GPIIIa: 0 = negative (reference), 1 = positive; ‡ART duration: duration of antiretroviral therapy, measured in months (continuous variable); #CD4 scaled: CD4+ T-cell count, with OR expressed per 100 cells/μL increase. Abbreviations: GPIIIa, glycoprotein IIIa (anti-platelet antibody); CI, confidence interval; OR, odds ratio; β, regression coefficient.

**Supplementary Table S9. Stratified sensitivity analysis: performance of the 4-variable thrombocytopenia prediction model in ART-treated and ART-naïve subgroups.**

**Part A. Comparison of multivariable logistic regression estimates across analytical subgroups: full cohort, ART-treated patients, and ART-naïve patients**

| Model | Variable | OR_95CI | P_value |
| --- | --- | --- | --- |
| Full cohort (n=196) | GPIIIa | 35.34 (9.21-135.67) | <0.001 |
| Full cohort (n=196) | CD4_scaled | 0.82 (0.68-0.97) | 0.033 |
| Full cohort (n=196) | Albumin | 0.91 (0.85-0.97) | 0.003 |
| Full cohort (n=196) | Neutrophils | 0.80 (0.68-0.92) | 0.004 |
| ART-treated only (n=157) | GPIIIa | 32.55 (9.44-162.44) | <0.001 |
| ART-treated only (n=157) | CD4_scaled† | 0.81 (0.66-0.98) | 0.045 |
| ART-treated only (n=157) | Albumin | 0.90 (0.83-0.97) | 0.006 |
| ART-treated only (n=157) | Neutrophils | 0.81 (0.67-0.95) | 0.014 |
| ART-naive only (n=39)‡ | GPIIIa | 241820010.28 (0.00-NA) | 0.994 |

OR: odds ratio; CI: confidence interval; ART: antiretroviral therapy. †CD4 scaled: CD4+ T-cell count, with OR expressed per 100 cells/μL increase.‡In the ART-naïve subgroup (n = 39), multivariable estimation was not feasible due to quasi-complete separation; the GPIIIa estimate is non-finite and should not be interpreted as a valid odds ratio. Univariable analysis only is reported for this subgroup.

**Part B. Area under the receiver operating characteristic curve (AUC) for the 4-variable model across analytical subgroups.**

| Model | AUC | CI_95 |
| --- | --- | --- |
| Full cohort (n=196) | 0.86 | 0.811-0.913 |
| ART-treated only (n=157) | 0.85 | 0.790-0.911 |
| ART-naive only (n=39)* |  | NA |

AUC: area under the receiver operating characteristic curve; CI: 95% confidence interval derived from DeLong method; ART: antiretroviral therapy. *AUC not computable for the ART-naïve subgroup due to quasi-complete separation in the multivariable model.Reference model: Full cohort (AUC = 0.862, 95% CI 0.811–0.913).

**Part C. Multivariable logistic regression for HIV-associated thrombocytopenia: stratified analysis restricted to ART-treated patients (n = 157).**

| term | estimate | std.error | statistic | p.value | conf.low | conf.high |
| --- | --- | --- | --- | --- | --- | --- |
| (Intercept) | 70.823 | 1.33300413 | 3.19593145 | 0.001394 | 5.846 | 1130.011 |
| GPIIIa† | 32.551 | 0.70773266 | 4.92109464 | 8.606E-07 | 9.439 | 162.439 |
| CD4_scaled# | 0.814 | 0.10249109 | -2.00875199 | 0.04456 | 0.657 | 0.985 |
| Albumin | 0.901 | 0.03752731 | -2.76463786 | 0.005699 | 0.834 | 0.967 |
| Neutrophils | 0.808 | 0.08707436 | -2.45185138 | 0.01421 | 0.671 | 0.946 |

†Anti-GPIIIa: 0 = negative (reference), 1 = positive; #CD4 scaled: CD4+ T-cell count, with OR expressed per 100 cells/μL increase. Abbreviations: ART, antiretroviral therapy; GPIIIa, glycoprotein IIIa (anti-platelet antibody); CI, confidence interval; OR, odds ratio; β, regression coefficient; SE, standard error.

**Part D. Exploratory univariable analysis in ART-naïve patients (n = 39): anti-GPIIIa antibody status.**

| term | estimate | std.error | statistic | p.value | conf.low | conf.high |
| --- | --- | --- | --- | --- | --- | --- |
| (Intercept) | 1.3 | 0.42062225 | 0.6237527 | 0.5328 | 0.572 | 3.045 |
| GPIIIa | 241820010 | 2688.50327 | 0.00718009 | 0.9943 | 0 |  |

Multivariable logistic regression could not be reliably fitted in this subgroup due to quasi-complete separation: anti-GPIIIa positivity near-perfectly discriminated thrombocytopenia outcomes within this small sample, yielding a non-finite multivariable coefficient estimate. The result shown represents the univariable association only and should be regarded as exploratory. Abbreviations: ART, antiretroviral therapy; GPIIIa, glycoprotein IIIa (anti-platelet antibody); CI, confidence interval; OR, odds ratio; SE, standard error.

**Supplementary Table S10. Sensitivity analysis incorporating age and sex: comparison of the primary 4-variable model and the age- and sex-adjusted model for predicting HIV-associated thrombocytopenia.**

**Part A. Multivariable logistic regression estimates: primary 4-variable model versus age- and sex-adjusted 6-variable model.**

| Model | Variable | OR (95% CI) | P-value |
| --- | --- | --- | --- |
| 4-variable (primary) | GPIIIa† | 35.344 (9.208-135.665) | <0.001 |
| 4-variable (primary) | CD4_scaled‡ | 0.821 (0.685-0.984) | 0.033 |
| 4-variable (primary) | ALB | 0.909 (0.854-0.968) | 0.003 |
| 4-variable (primary) | Neutrophils | 0.798 (0.683-0.932) | 0.004 |
| +Age+Sex (sensitivity) | GPIIIa† | 35.194 (9.048-136.898) | <0.001 |
| +Age+Sex (sensitivity) | CD4_scaled‡ | 0.824 (0.688-0.987) | 0.036 |
| +Age+Sex (sensitivity) | ALB | 0.909 (0.853-0.968) | 0.003 |
| +Age+Sex (sensitivity) | Neutrophils | 0.800 (0.684-0.936) | 0.005 |
| +Age+Sex (sensitivity) | Age* | 0.989 (0.961-1.018) | 0.452 |
| +Age+Sex (sensitivity) | Sex# | 1.101 (0.461-2.626) | 0.829 |

†Anti-GPIIIa: 0 = negative (reference), 1 = positive; ‡CD4 scaled: CD4+ T-cell count, with OR expressed per 100 cells/μL increase; #Sex: female = reference category, male vs. female (binary variable); *Age: continuous variable, OR expressed per 1-year increase. Abbreviations: GPIIIa, glycoprotein IIIa (anti-platelet antibody); ALB, albumin; CI, confidence interval; OR, odds ratio.

**Part B. Model discrimination: area under the receiver operating characteristic curve (AUC) for the primary and age- and sex-adjusted models.**

| Model | AUC | CI_95 | DeLong_P |
| --- | --- | --- | --- |
| 4-variable (primary) | 0.862 | 0.811-0.913 | Reference |
| +Age+Sex (sensitivity) | 0.864 | 0.813-0.914 | 0.5285 |

AUC: area under the receiver operating characteristic curve; CI: 95% confidence interval derived from the DeLong method; DeLong P: P-value from DeLong test comparing AUC between models.Reference model: 4-variable primary model (GPIIIa, CD4 count, albumin, neutrophils).A DeLong P > 0.05 indicates no statistically significant difference in discriminative ability between the two models.

**Supplementary Table S11. Logistic regression analysis of thrombocytopenia risk by CD4 count and anti-GPIIIa antibody status**

**Part A: Model comparison by antibody status**

| Model | N | N_Cases | Prevalence | CD4_Coefficient | CD4_OR_per_unit | CD4_OR_per_100 | CD4_PValue | AIC |
| --- | --- | --- | --- | --- | --- | --- | --- | --- |
| Full Model | 167 | 92 | 55.1% | -0.003446 | 0.996560 | 0.7085 | P = 0.008 | 178.61 |
| Anti-GPIIIa Negative | 119 | 47 | 39.5% | -0.003446 | 0.996560 | 0.7085 | P = 0.008 | 155.91 |
| Anti-GPIIIa Positive | 48 | 45 | 93.8% | -0.006435 | 0.993586 | 0.5255 | P = 0.069 | 22.70 |

**Part B: Interaction analysis**

| Effect | Coefficient | SE | Z_value | P_Value | Interpretation |
| --- | --- | --- | --- | --- | --- |
| Anti-GPIIIa Main Effect | 3.923601 | 1.343258 | 2.921 | P = 0.003 | Significant |
| CD4 Main Effect | -0.003446 | 0.001292 | -2.667 | P = 0.008 | Significant |
| Interaction (CD4 × GPIIIa) | -0.002989 | 0.003762 | -0.795 | P = 0.427 | Not significant |

CD4 coefficient represents change per 1 cell/μL; OR per 100 cells/μL calculated as exp(β × 100). The non-significant interaction term (*P* = 0.427) indicates that CD4 effects do not differ significantly between antibody groups, although absolute risk levels remain markedly different (>50 percentage points) across all CD4 strata.
